# Identification and comparative analysis of the epidermal differentiation complex in snakes

**DOI:** 10.1038/srep45338

**Published:** 2017-03-27

**Authors:** Karin Brigit Holthaus, Veronika Mlitz, Bettina Strasser, Erwin Tschachler, Lorenzo Alibardi, Leopold Eckhart

**Affiliations:** 1 Research Division of Biology and Pathobiology of the Skin, Department of Dermatology, Medical University of Vienna, Vienna, Austria; 2 Dipartimento di Scienze Biologiche, Geologiche ed Ambientali (BiGeA), University of Bologna, Bologna, Italy

## Abstract

The epidermis of snakes efficiently protects against dehydration and mechanical stress. However, only few proteins of the epidermal barrier to the environment have so far been identified in snakes. Here, we determined the organization of the Epidermal Differentiation Complex (EDC), a cluster of genes encoding protein constituents of cornified epidermal structures, in snakes and compared it to the EDCs of other squamates and non-squamate reptiles. The EDC of snakes displays shared synteny with that of the green anole lizard, including the presence of a cluster of corneous beta-protein (CBP)/beta-keratin genes. We found that a unique CBP comprising 4 putative beta-sheets and multiple cysteine-rich EDC proteins are conserved in all snakes and other squamates investigated. Comparative genomics of squamates suggests that the evolution of snakes was associated with a gene duplication generating two isoforms of the S100 fused-type protein, scaffoldin, the origin of distinct snake-specific EDC genes, and the loss of other genes that were present in the EDC of the last common ancestor of snakes and lizards. Taken together, our results provide new insights into the evolution of the skin in squamates and a basis for the characterization of the molecular composition of the epidermis in snakes.

Snakes are reptiles that have lost their limbs during evolution and developed a unique predatory lifestyle that involves the ability to swallow prey of a diameter larger than that of their own body^1^,^2^,^3^. The skin of snakes, and more specifically the epidermis, consists of rigid scales and soft inter-scale regions, which together provide both mechanical resistance and flexibility^4^,^5^. Snakes belong to the squamate reptiles which are characterized by the regular shedding of the outer layers of the epidermis, also known as ecdysis^6^,^7^. In snakes, the superficial layers of the epidermis are detached as a single, coherent sheet whereas other squamates (lizards and geckos) shed multiple smaller flakes. While the dynamic regulation and composition of snake skin has been partially revealed over the past fifty years^7^,^8^,^9^, the recent availability of whole genome sequences of snakes and other reptiles allows, for the first time, to identify genes that encode epidermal proteins and, by comparative genomics, to establish a basis for building hypotheses on the molecular evolution of the epidermis in snakes^10^,^11^,^12^.

In all amniotes, keratinocytes proliferate in the basal layer and differentiate in the suprabasal layers of the epidermis^13^,^14^. Signaling between the epidermis and the underlying dermis controls the patterning of the epidermis and the formation of skin appendages^15^,^16^,^17^,^18^. While differentiation of keratinocytes in the mammalian epidermis involves continuous alterations of cell structures and movement of cells towards the body surface, keratinocyte differentiation in squamates results in the formation of distinct non-interconvertible layers that remain stable for several weeks before they are shed together^8^,^9^. In its final differentiation stage, the outer generation of the epidermis comprises a clear, lacunar, alpha, mesos, beta, and oberhautchen layer^7^,^8^,^9^. Prior to shedding, the outer generation of the epidermis protects the resting and newly forming inner generation of the epidermis. At the beginning of skin development, the cell layers of the embryo-specific periderm cover the epidermis^19^. The histological and ultrastructural features of squamate skin have been reported^7^,^8^,^9^,^20^. However, only few aspects of the molecular architecture of alpha and beta-layers of squamate epidermis have been determined so far^21^,^22^,^23^,^24^.

Corneous beta-proteins, traditionally called beta-keratins, have been identified as components of the epidermis in snakes, like in other reptiles^22^,^25^,^26^,^27^. Keratin intermediate filament proteins, previously referred to as alpha-keratins, are the main cytoskeletal proteins in the epidermis and skin appendages of vertebrates. A cysteine-rich keratin component of reptilian claws has been lost due to gene inactivation during the evolution of snakes^28^. The presence of various ultrastructurally, but not biochemically, defined epidermal components such as fibers in the beta-layer^29^ and different granules in the oberhautchen and the clear layer of reptiles^21^ indicate that many structural proteins of snake epidermis remain to be identified.

The recent availability of genome and transcriptome sequences from multiple vertebrates has allowed the determination of genes implicated in epidermal structure and function. Based on the dermatologically relevant characterization of human epidermal barrier genes, we have screened non-mammalian tetrapods for homologs of a gene cluster known as the Epidermal Differentiation Complex (EDC)^30^,^31^,^32^,^33^,^34^. Genes encoding S100 fused-type proteins (SFTPs), which are homologous to a subgroup of mammalian EDC genes^35^, were found in amphibians^34^, and more complex gene clusters homologous to the mammalian EDC were identified in the chicken^30^,^32^, in the green anole lizard (*Anolis carolinensis*)^30^ and in turtles^33^.

Here we extend the comparative analysis of the EDC in sauropsids and determine the gene complement of the EDC in snakes. We characterize the amino acid sequences of EDC-encoded proteins, suggest hypotheses about their contributions to the molecular architecture of the epidermis, and identify cases of gain and loss of specific EDC genes during the evolution of stem lepidosaurs and snakes.

## Results

### Identification of epidermal differentiation complex (EDC) genes in snake genomes

The EDCs of the Burmese python (*Python bivittatus*) and the king cobra (*Ophiophagus hannah*) were defined as the genomic regions flanked by S100A genes, like in other amniotes^30^,^33^. The gene complement of the EDC of these snakes was identified by tBLASTn searches using EDC-encoded proteins of *A. carolinensis*, chicken and humans as queries and by *de novo* prediction of genes in an iterative process, as described previously^33^. The predicted amino acid sequences of snake EDC proteins were used as queries in tBLASTn searches in the published transcriptomes of snakes to test for the expression of the predicted genes.

The nomenclature for EDC genes follows the preliminary system defined in previous studies^30^. In brief, gene names consist of the term Epidermal Differentiation (ED) followed by a term that describes the amino acid composition or the presence of particular amino acid sequence motifs in the encoded protein. For easier readability, only the abbreviations are used in the text whereas the full names of genes are summarized in Supplementary Table S1. Exceptions to this naming convention were made to indicate orthologs of human loricrin and cornulin and chicken scaffoldin.

### The EDC of snakes is largely syntenic with that of the green anole lizard

**Figure 1:**
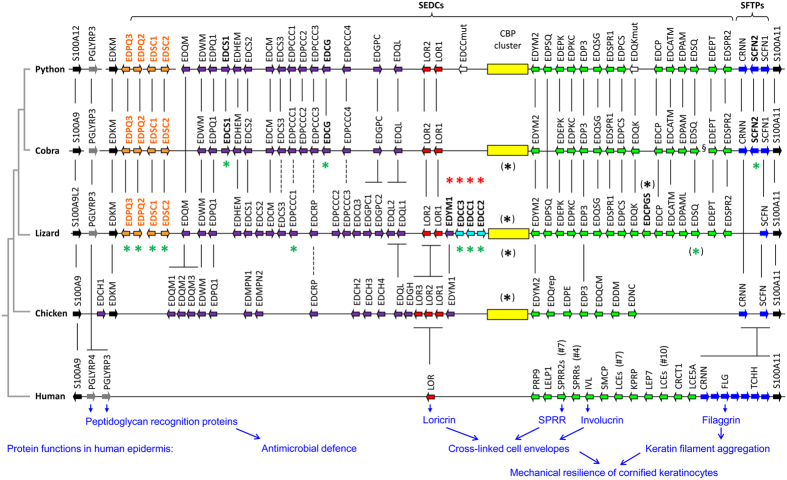
Organization of the epidermal differentiation complex (EDC) in the Burmese python and the king cobra. Genes of the EDC in snakes *Python bivittatus* and *Ophiophagus hannah*, in comparison to those of the lizard (*Anolis carolinensis*), the chicken, and human, are schematically depicted. Arrows indicate the orientation of the genes. Simple EDC (SEDC) genes with 2 exons are represented by colored arrows with a black frame whereas other genes are shown as filled arrows. Corneous beta-protein (CBP) gene clusters are shown as boxes in this diagram while detailed information about the genes in these clusters are depicted in Fig. 2. Members of gene families are numbered according to the positions of genes without indicating 1:1 orthology to specific members of the same gene family in other species. The depiction of the human EDC is simplified by representing gene family clusters with arrows and indicating the total number (#) of genes within each cluster. Black vertical lines connect orthologous genes or gene families. Green and red asterisks indicate putative gene gain and loss events whereas black asterisks indicate gene differences that could not be unambiguously assigned to an evolutionary event in a particular lineage. Note that the diagram is not drawn to scale. The symbol § marks a locus in which genes unrelated to classical EDC genes are present in the current genome sequence assembly of the cobra. Because of improved delineation of orthology relationships, the following gene names have been newly assigned to replace previous names^30^: lizard EDQL instead of EDCQ, chicken EDQM3 instead of EDSC, and EDPQ1 instead of EDCH5.

The structure of the EDC is very similar in the Burmese python, the king cobra, and the green anole lizard (Fig. 1). Like in other amniotes^30^,^33^, the EDC of snakes is bordered by S100A genes and comprises a peptidoglycan recognition protein 3 (*PGLYRP3*) gene, simple (single coding exon) EDC (SEDC) genes, and SFTP genes (Suppl. Tables S2-6; Suppl. Figures S1 and S2). A gene homologous to *EDKM* of lizards, turtles and birds is located between the *PGLYRP3* and the SEDC genes (Fig. 1). In the draft genome of the king cobra, the genes *EDSQ* and *EDEPT*, the orthologs of which are neighbors in the python genome, were separated by a series of genes not related to the classical EDC genes and a sequence gap. This pattern indicates that a gene rearrangement event might have disrupted the canonical organization of the EDC in this species (Fig. 1, §).

To test whether the predicted EDC genes of snakes are expressed, tissue transcriptomes of snakes were screened by tBLASTn searches. Indeed, transcript reads corresponding to most EDC genes (Suppl. Tables S2 and S3), were detected in skin transcriptomes of the ball python (*Python regius*)^36^ (Suppl. Fig. S3) and of the painted saw-scaled viper (*Echis coloratus*)^37^, whereas the transcriptomes of internal organs of snakes included no or only very small numbers of EDC gene transcripts (Suppl. Fig. S4), suggesting a skin-specific expression of most EDC genes.

### A unique corneous beta-protein (CBP) comprising 4 beta-sheets is conserved in squamates

**Figure 2:**
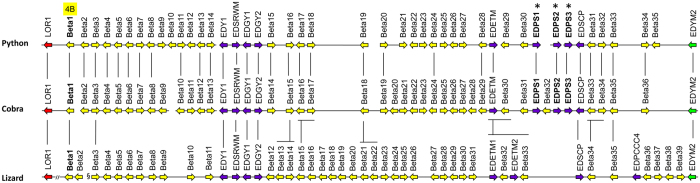
Organization of the corneous beta-protein (CBP), also known as beta-keratin, gene cluster in snakes. The CBP gene clusters in python (*P. bivittatus*), cobra (*O. hannah*) and lizard (*A. carolinensis*) are schematically depicted. The CBP genes were tentatively named “Beta” followed by a number that indicates the position in the cluster. The CBP genes of the lizard correspond to the “Li-Ac” genes reported previously^26^, whereby Beta1 and 2 are identical to Li-Ac40 and 39, respectively, and Beta3 through 39 are identical to Li-Ac37 through 1. A gap in the lizard genome sequence assembly (§) is the likely locus of Beta40 (Li-Ac38). Arrows indicate the orientation of the genes. CBP genes are represented by yellow arrows whereas non-CBP genes inside the cluster are shown by violet arrows. The label “4B” marks the presence of 4 beta-sheets in the encoded protein (Fig. 3) and asterisks indicate snake-specific genes (Suppl. Fig. S8). *LOR1* and *EDYM2* are conserved genes flanking the CBP gene cluster. Vertical lines indicate orthologs. Note that the schemes are not drawn to scale.

SEDC genes form a continuous cluster in snakes and include a sub-cluster of genes that encode corneous beta-proteins (CBPs), also known as beta-keratins^14^. These proteins are characterized by a conserved core domain that is predicted to form a beta-sheet^38^. The CBP cluster gene is located between the *loricrin* and *EDYM2* genes of snakes (Fig. 1, Fig. 2). It is syntenic with the CBP locus of the green anole lizard and with the main CBP loci of birds and turtles^30^,^33^. Within the CBP gene cluster, 35 and 36 CBP genes, here termed *Beta1* through *Beta36* in order of the arrangement of the genes, were identified in the python and cobra, respectively, which is comparable to the 40 CBP genes present in the green anole lizard^26^, but lower than the 71 CBP genes reported for the Japanese gecko^39^.

**Figure 3:**
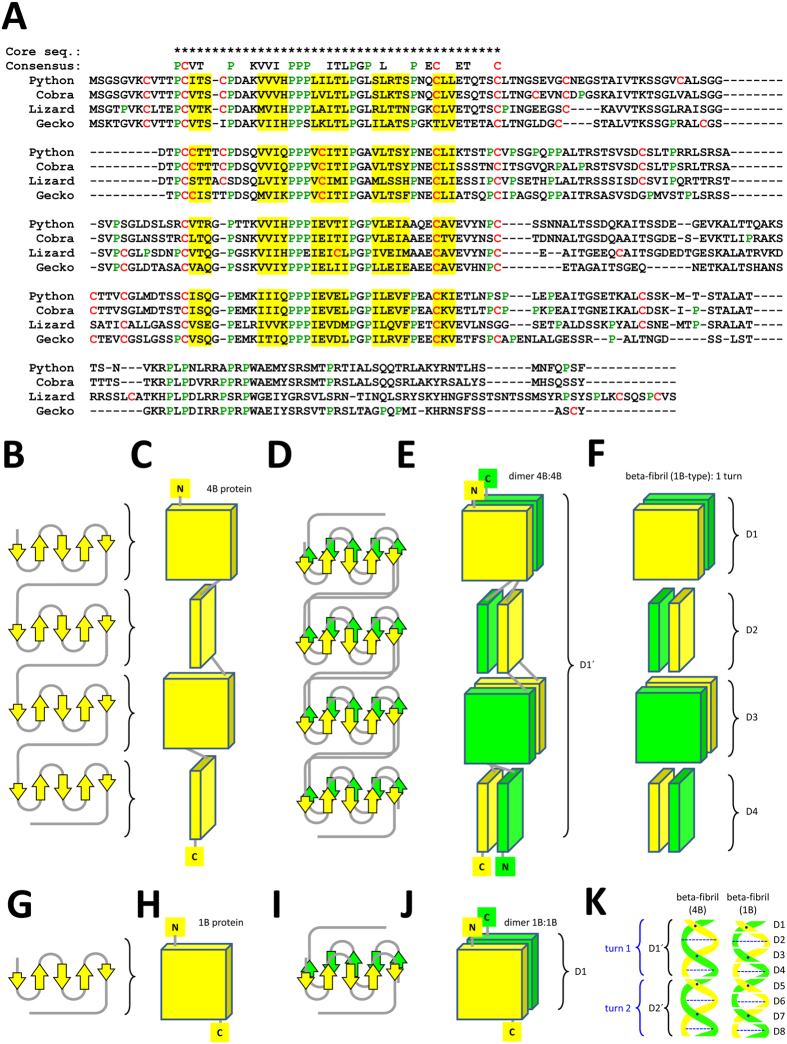
A corneous beta-protein with 4 beta-core sequence motifs is predicted to facilitate the formation of unique beta-fibrils in squamates. (**A**) Amino acid sequence alignment of Beta1 proteins of python (*P. bivittatus*), cobra (*O. hannah*), lizard (*A. carolinensis*) and gecko (*G. japonicus*). Internal sequence repeats were aligned to illustrate the conservation of segments corresponding to the CBP core sequences (indicated by * above the alignment) (see also Suppl. Fig. S5). Key residues of the repeat consensus sequence are indicated. Putative beta-strand-forming residues are indicated by yellow shading. Proline and cysteine residues are shown in green and red fonts, respectively. (**B–K**) Modeling of protein structures. The model for the folding (**B**,**C**) and dimerization (**D**,**E**) of CBPs with 4 beta-sheets (B4 proteins) was adapted from the model for CBPs with 1 beta-sheet (B1 proteins) (**G–J**) which also proposes a helical arrangement of 4 CBP dimers in 1 turn of the helical structure of a beta-fibril^41^ (**F**). The model for B4 proteins suggests that a dimer (D1′) (**E**) is equivalent to 1 turn of a beta-fibril (**F**) and 2 B4-protein dimers (D1′ and D2′) substitute for 8 B1-protein dimers to form 2 turns of a beta-fibril helix (**K**). The orientation of the dimerization interfaces is indicated by dashed lines and dots in (**K**). Note that the schematic depiction of beta-sheets is simplified and does not show a twist typically present in beta-sheets. C, carboxy-terminus; N, amino-terminus.

Both the python and cobra have an ortholog of the lizard gene *Beta1*, previously termed *Li-Ac40*^26^ (Fig. 2). This gene encodes a protein that contains 4 CBP core sequence motifs and therefore is predicted to form 4 beta-sheets (Fig. 3; Suppl. Fig. S5) whereas all other CBPs identified so far comprise only a single beta-sheet domain. Remarkably, classical CBPs undergo dimerization via face-to-face interactions between their beta-sheets and subsequently they assemble into a beta-fibril (beta-filament) in which, according to the classical model^38^,^40^,^41^, 4 dimers form one turn of the helical structure. We put forward a hypothetical model in which 2 Beta1 proteins dimerize via their 4 beta-sheets and thereby form one complete turn of the helical structure of a beta-fibril (Fig. 3). The integration of Beta1 dimers into fibrils likely occurs via edge-to-edge interactions in a manner equivalent to that proposed for dimers of CBPs with 1 beta-sheet domain^41^,^42^. As there are currently no experimental data on sauropsidian CBPs, that could resolve the structure of beta-fibrils at atomic resolution^41^, the integration of the complete Beta1 sequences of squamates into ongoing computer modelling attempts^42^ will extend the scope of these studies beyond the investigation of interactions between isolated beta-sheets. As Beta1 homologs are present in snakes, lizards, and geckos (Fig. 3), but not in other sauropsids, these proteins represent an evolutionary innovation of squamates.

Interestingly, several genes within the CBP locus encode proteins that lack a beta-sheet-forming domain, i.e. the defining feature of CBPs, but share the exon-intron organization with CBP and other SEDC genes (Fig. 2, Suppl. Fig. S1 and S2). The positions of these non-CBP genes relative to specific *CBP*s are largely conserved between snakes and the green anole lizard (Fig. 2). BLAST searches with the sequences of newly identified EDC genes allowed us to identify previously uncharacterized homologs of these genes in the green anole lizard (Suppl. Table S6; Suppl. Fig. S6).

### The EDC of squamates contains multiple genes that encode proteins with extremely high cysteine contents

The amino acid sequences of snake EDC proteins were analyzed for the presence of conserved sequence motifs. Snake S100A genes and SFTPs contain an amino-terminal S100 domain^43^ while snake PGLYRP3 is predicted to acquire the characteristic structural fold also found in other PGLYRPs^43^. Among SEDC proteins, only CBPs contain a structural motif, *i.e.* the beta-sheet forming region, whereas other SEDCs of snakes do not contain sequences associated with the propensity to fold into a known protein domain. However, many snake SEDC proteins have amino-terminal and carboxy-terminal sequence motifs that are conserved in SEDCs of a diverse range of amniotes, including humans (Suppl. Fig. S7).

**Figure 4:**
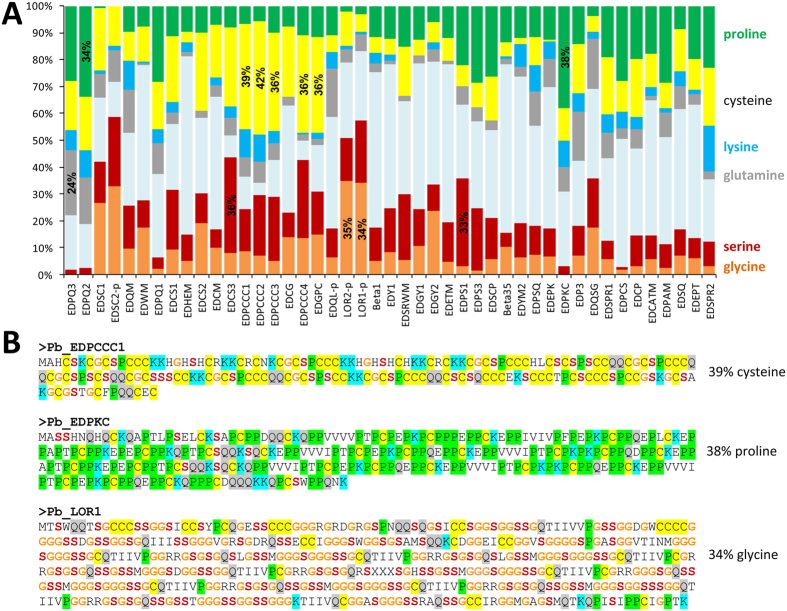
SEDC genes encode proteins with extremely biased amino acid composition. (**A**) The diagram shows the amino acid compositions of SEDC proteins of *Python bivittatus*. The protein data are shown in the order of the corresponding genes in the EDC (Fig. 1). Translation products of genes within the corneous beta-protein (CBP) gene cluster (Fig. 2) are not included here, with the exception of proteins encoded by the first and the last CBP. (**B**) Amino acid sequences of exemplary SEDC proteins of the *P. bivittatus*.

SEDC proteins of amniotes are generally characterized by amino acid sequence repeats and high abundance of one or several of the following amino acid residues: glycine (G), serine (S), proline (P), glutamine (Q), and cysteine (C)^30^,^33^. Likewise, the EDC of the python comprises genes that encode proteins with high contents of G (e.g., loricrins 1 and 2, > 34% G), S (EDCS3, 36% S; EDPS1, 33% S), P (EDPQ2, 34% P; EDPCK, 38% P) and Q (EDPQ2, 24% Q) (Fig. 4 and Suppl. Fig. S1). Cysteine-rich amino acid sequences are encoded by many SEDC genes of the snakes, which is surprising in the context of the hypothesis that cysteine-rich proteins function mainly in hard and resistant skin appendages such as claws, hair and feathers, and tend to be lost in species, such as snakes, that lack these skin appendages^28^.

Fifteen EDC genes of the python encode proteins containing at least 20% cysteine, and among them 5 proteins have a cysteine content above 35% (Fig. 4B). Five python EDC genes encoding proteins with high cysteine content were clustered between *EDPQ1* and *EDQL*, i.e. two genes evolutionarily conserved in lizard and chicken (Fig. 1). This region of the python EDC is syntenic with the locus of a gene encoding the cysteine-rich feather protein (EDCRP) in the chicken^32^, while the homologous locus of the green anole lizard contains a gene encoding an EDCRP-like protein^32^ and 9 other cysteine-rich proteins (EDCS1, EDCS3, EDPCCC1-4, EDGPC1-2 and EDCQ3). Among the latter, 8 were identified in the present study (Fig. 1; Suppl. Fig. S6). The highest cysteine contents so far detected among squamate EDC proteins are present in *A. carolinensis* EDPCCC4 (45% cysteine) and EDPCCC1 of the king cobra (46.6% cysteine). EDPCCC1 is conserved among snakes, and by the analysis of tissue transcriptomes of *E. coloratus* we could confirm expression in the skin whereas internal organs lacked EDPCCC1 (Suppl. Fig. S4C). The cysteine-rich proteins of snakes and other squamates are characterized by repetitive amino acid sequences with clusters of two or more cysteine residues (Fig. 4B). These proteins are candidates to become cross-linked components of the hard scales of squamates but this hypothesis remains to be tested in future studies.

### Identification of snake-specific EDC gene innovations and losses

Differences in the EDCs of snakes and the anole lizard (Fig. 1) suggest that gene innovations or gene losses have occurred in either one of the evolutionary lineages leading to snakes and iguanians (represented here by *A. carolinensis*). To determine the ancestral condition for each of the clade-specific genes, we searched for orthologous genes in the Japanese gecko (*G. japonicus*), representing Gekkota, a basal clade of squamates and the closest outgroup to Toxicofera^44^,^45^,^46^ with a sequenced genome^39^. These comparisons suggested that some EDC genes have been lost in snakes whereas others have originated in the snake lineage.

**Figure 5:**
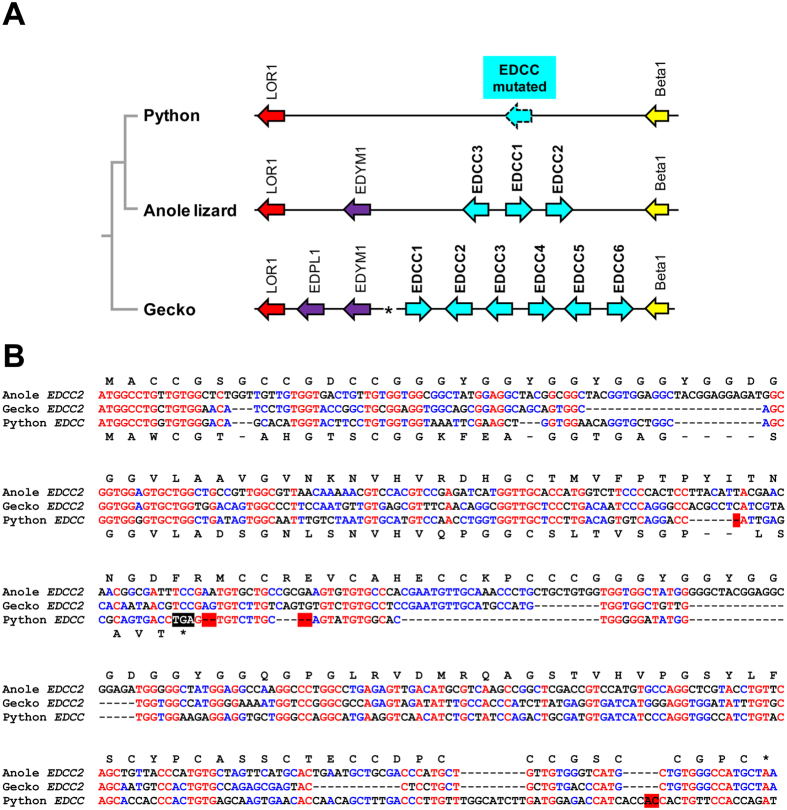
EDCC genes have accumulated inactivating mutations in snakes. (**A)** The gene loci of EDCC and EDYM1 genes in the green anole lizard (Ac, *Anolis carolinensis*) and the gecko (Gj, *Gekko japonicus*) were compared to that of the python (Pb, *Python bivittatus*). (**B**) Nucleotide sequence alignment of the mutated EDCC sequence of the python and EDCC genes of the anole lizard and the gecko. Red fonts indicate conservation in all 3 sequences and blue fonts indicate conservation in 2 of 3 sequences. Red shading indicates frame-shift mutations and a premature stop codon in the python genes is shown with white fonts on black background. Amino acid sequences obtained by in silico translation of anole and python genes are shown above and below the nucleotide sequence alignment, respectively.

Genes of the EDCC family (Fig. 5A), which encode proteins rich in cysteine-cysteine motifs, are present in the Japanese gecko (n = 6) and in the green anole lizard (n = 3) but not in snakes (a sister group of the green anole lizard). This species distribution of EDCCs suggests that EDCC gene(s) were present in the last common ancestor of snakes and lizards and later underwent inactivation in snakes. In agreement with this hypothesis, we identified a mutated remnant of an EDCC gene located between *loricrin 1* and the CBP gene cluster of the python (Fig. 5B). Orthologs of EDCC are absent from the EDCs of birds and turtles^30^,^33^, indicating that EDCCs represent a squamate-specific gene innovation. The gene *EDYM1*, which is conserved in turtles and birds^33^, is located between *EDCC* and *loricrin* genes in the anole lizard and the gecko whereas it is absent in snakes (Fig. 1). These data suggest that *EDYM1* and the *EDCC* genes of the last common ancestor of Toxicofera (snakes and iguanid lizards) were lost in snakes.

A group of apparently snake-specific proteins are encoded by the genes *EDPS1* through *EDPS3 (epidermal differentiation proteins rich in proline and serine 1-3*) (Suppl. Fig. S8), which are located within the CBP gene cluster of snakes but not in the CBP cluster of other squamates (Fig. 2). The investigation of tissue transcriptomes of *E. coloratus* suggested that EDPS homologs are expressed in the skin but not in internal organs (Suppl. Fig. S4E).

### Duplication of scaffoldin in snakes

**Figure 6:**
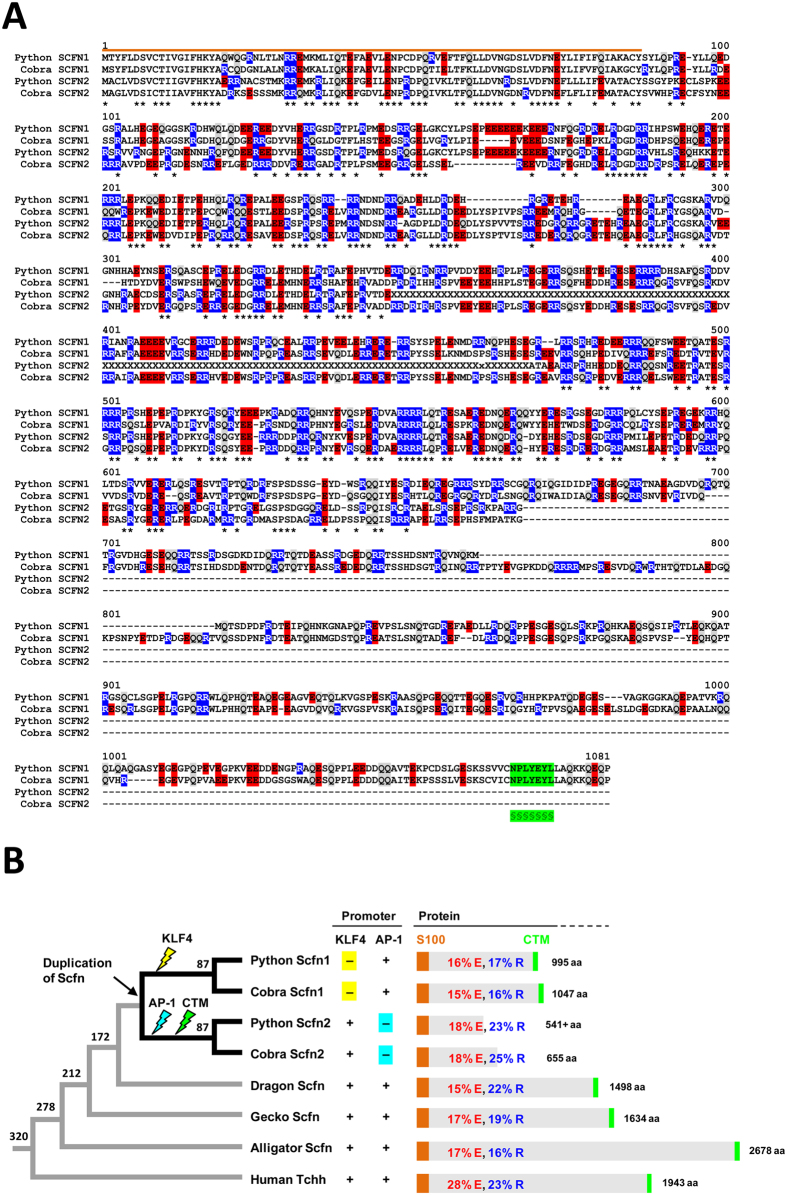
Duplication and sequence diversification of scaffoldin in snakes. (**A**) Amino acid sequence alignment of scaffoldin (SCFN) 1 and 2 proteins of python and cobra. The S100 domain is indicated by an orange-colored line above the alignment. A carboxy-terminal motif (CTM) (§) implicated in keratin binding is highlighted by green shading. The predominant amino acid residues, i.e. glutamic acid (E) and arginine (R), are highlighted by red and blue shading. Positions with identical amino acid residues in all 4 sequences are indicated by asterisks below the alignment. (**B**) Schematic phylogenetic tree for SFTPs in amniotes. Numbers at the branching points of the species tree indicate the divergence times (million years ago) of phylogenetic lineages. The presence of putative binding sites for the transcription factors KLF4 and AP-1 in the promoters (Suppl. Fig. S10) are indicated. The organization of the proteins is depicted schematically with orange boxes indicating the S100 domain and green boxes indicating the CTM. The contents of glutamate (E) and arginine (R) of each protein are shown. aa, amino acids; Tchh, trichohyalin.

Snakes have 3 SFTP genes (*Crnn, Scfn1, Scfn2*) (Fig. 6) whereas the green anole lizard has only one (*Scfn*) while the bearded dragon, the Japanese gecko, the American alligator and the chicken have two (*Crnn, Scfn*)^30^,^31^. Both isoforms of snake scaffoldin proteins are rich in glutamic acid (E) and arginine (R) residues (Fig. 6A), which are also highly abundant in human trichohyalin but not in cornulin^31^. Expression of both *Scfn* genes was confirmed by intron-spanning RNA-seq reads in the painted saw-scaled viper (*Echis coloratus*) (Suppl. Fig. S9).

Phylogenetic profiling and gene locus comparison suggested that the *Scfn2* gene originated by duplication of the primordial *Scfn* gene specifically in snakes (Fig. 6B). The sequences of the proximal promoters of *Scfn1* and *Scfn2* genes were partly conserved, and they contained homologous TATA boxes. Remarkably, the putative binding sites for 2 transcription factors (KLF4 and AP-1) in promoters of SFTP genes^31^ were differentially conserved in snake *Scfn1* and *Scfn2* (Fig. 6B, Suppl. Fig. S10). The predicted binding site for AP-1 was present in the promoters of *Scfn1* but not of *Scfn2* genes whereas the KLF4 binding site was present in the promoters of *Scfn2* but not of *Scfn1* genes of both python and cobra. Taken together, these data suggest a scenario in which a single ancestral *Scfn* gene was present in the last common ancestor of snakes and the green anole lizard, this gene was duplicated in primitive snakes, and the derived genes underwent divergent evolution of their promoter sequences.

Moreover, the SCFN2 proteins lack a carboxy-terminal sequence motif (CTM) that is present in SCFN1 of snakes and in most other SFTPs of amniotes^31^,^47^ (Fig. 6). As this motif has been implicated in keratin filament binding of SFTPs^47^, divergent amino acid sequence evolution appears to have caused also differences in the functions of scaffoldins 1 and 2 of snakes.

## Discussion

The results of the present study shed new light on the molecular composition and evolution of the epidermis in snakes and other squamates. The comparative analysis of snake EDCs suggests that the epidermis of snakes contains many more proteins than the small set of CBPs (beta-keratins) identified in previous studies^25^,^26^. Both the number of CBPs and the number of other EDC genes of snakes is similar to those of the green anole lizard, and the total number and sequence diversification of epidermal differentiation genes (including CBPs) in snakes exceeds that present in mammals (Fig. 1). The new data therefore indicate that the process of keratinocyte cornification in the epidermis of snakes requires the participation of numerous proteins aside CBPs, like in other sauropsids. While crucial roles of mammalian EDC genes such as loricrin, LCEs, trichohyalin, and filaggrin in the skin barrier of mammals have been defined by a long series of studies^48^,^49^,^50^,^51^,^52^, the identification of the EDC gene complement of snakes is the pivotal starting point for a comprehensive investigation of epidermal differentiation in this important subgroup of reptiles.

Important limitations of this study were the quality of the genome sequences that were available for analysis and the focus of our study on genes of the EDC. The current genome sequences of squamates are not of the same quality as those of mammalian model species and, therefore, some gaps are present in our model of the EDC in snakes (Fig. 1). The aim of this study was the characterization of the EDC in snakes; and other genome loci that control distinct aspects of epidermal differentiation, such as the enzymatic control of protein cross-linking during keratinocyte cornification and the disruption of intercellular junctions during ecdysis have not been investigated here.

The EDC of snakes is largely syntenic with that of the green anole lizard, and only few genes are not orthologous between the two taxa. As snakes lack the specialized epidermal differentiation pathways that lead to the formation of claws and toe pad lamellae, the large degree of EDC gene conservation between snakes and the anole lizard suggests that the great majority of EDC genes play essential roles in skin structures unrelated to limb-specific appendages. Nevertheless, the present identification of several snake-specific changes in the EDC gene complement (loss of EDCCs and EDYM1, origin of EDPS, duplication of scaffoldin) points to unique characteristics of the epidermal differentiation in snakes, likely evolved during their specific adaptation to their environment. EDCCs contain multiple CC dipeptides and stretches of proline residues, respectively, and these proteins show limited similarity with other SEDC proteins, indicating that they have non-redundant roles. Likewise, the amino acid sequences of scaffoldins 1 and 2, as well as their promoters, differ substantially. Recently, we have shown that scaffoldin of the chicken is expressed in the embryonic periderm and in epithelial cells that support the morphogenesis of claws and feathers by providing a transient epithelial scaffold which degenerates after maturation of these skin appendages^31^. These data contributed to the evolutionary-developmental model that connects the embryonic archosaur scale and feathers^16^,^17^,^18^,^53^. The identification of the genes encoding scaffoldins 1 and 2 will facilitate the determination of their expression during embryonic development and during the shedding cycle of snakes in future studies. Likewise, the future investigation of other EDC genes, that have either been lost or acquired specifically in the snake lineages, will help to elucidate differences between the epidermis of snakes and other squamates.

The presence of multiple genes encoding cysteine-rich EDC proteins in snakes suggests that a high cysteine content of proteins is not only required for hard skin appendages such as claws, hair and feathers. These skin appendages consist of entirely cornified proteinaceous structures and are absent in snakes. Hard skin appendages utilize cysteine-dependent disulfide protein cross-linking to acquire high mechanical resilience. In mammals, cysteine-rich keratins are components of hair and nails whereas keratins with low cysteine content are components of the soft epidermis^54^. Cysteine-rich keratin-associated proteins (Krtaps) are further components of mammalian hair and nails, and a cysteine-rich EDC protein (EDCRP) is a component of avian feathers^30^,^32^. Our detection of multiple EDC genes for high-cysteine proteins in snakes suggests that these epidermal proteins can have functions unrelated to claws, hair and feathers. Pythons and boas have spurs (rudimentary claws) that are located next to their cloaca, but other snakes do not have homologs of claws. Thus, disulfide bond-mediated cross-linking of cysteine-rich proteins may contribute to the maturation of hard scales in snakes and probably also in other squamates. In this regard, it will be interesting to compare the expression pattern of cysteine-rich EDC proteins in different types of snake scales, i.e. flat and tough scales on the head, keeled and perhaps softer scales on the dorsum and the sides of the body, and large, mechanically resistant ventral scales (gastrosteges) that are utilized for movement. However, the presence of high cysteine contents in the absence of hard skin appendages may also point to a role of cysteine residues that is unrelated to disulfide bond formation. Cysteine residues have been identified as attachment sites for palmitic acid which allows the anchoring of proteins into membranes^55^. The process of cysteine palmitoylation has been demonstrated in mammalian skin proteins, but whether a similar process also occurs in snakes requires further investigations.

Our finding that a unique CBP (beta-keratin) comprising 4 beta-sheets is conserved among and specific for squamates indicates that this protein contributes to unique properties of epidermal keratinocytes in squamates. Previous immunolabeling studies in the green anole lizard have suggested expression of Beta1, also referred to as Li-Ac40, in the beta-layer of scales on different body sites investigated^56^. The immunolabeling for this large beta-protein was associated with filaments of 3 nm thickness, i.e. the characteristic diameter of beta-fibrils. The classical model of the beta-fibril structure was developed more than 30 years ago on the basis of X-ray diffraction studies^38^,^40^. According to this model, 4 CBP (then called beta-keratin) dimers form a turn of a left-handed helix with four repeating units per turn. It is now striking that the only CBP comprising more than 1 beta-sheet-forming segment contains 4 such elements, indicating that it may span exactly 1 turn of the fibril. Of note, an alternative model in which 4 CBP dimers would be arranged to form half of a helix turn has also been reported^42^. Many aspects of beta-fibril formation in squamates and sauropsids in general are still open and comparative studies on CBPs with 1 and 4 beta-sheet regions may yield valuable insights in future studies.

In conclusion, the results of this study establish a comprehensive catalog of EDC genes of snakes and, thereby, provide the basis for further studies on the molecular organization and evolution of the epidermis in snakes and other squamates.

## Methods

### Genome sequences and gene identification

Genome sequences from the following squamate species were used for gene predictions: Burmese python (*Python bivittatus*)^10^, king cobra (*O. hannah*)^11^, painted saw-scaled viper (*Echis coloratus*)^36^, bearded dragon (*Pogona vitticeps*)^57^ and the Japanese gecko (*Gekko japonicus*)^39^. The accession numbers of genome sequence scaffolds corresponding to the EDC are listed in Supplementary Tables S2 through S6. Coding sequences of EDC genes were predicted using a combination of the following approaches. Amino acid sequences of EDC proteins of *A. carolinensis*^30^ and humans^35^ were used as queries in tBLASTn searches against the nucleotide sequence between *S100A12* and *S100A11* genes of the target genome. Information about exon coverage by RNA-seq reads, available in the NCBI browser for “genomic regions, transcripts, and products”, was used to identify transcribed regions in the EDC of *P. bivittatus*. The transcribed regions were translated and the resulting amino acid sequences were compared to those of known EDC proteins. The nucleotide sequence of EDC regions of apparently low gene density was translated *in silico*, and additional open reading frames of candidate EDC genes were identified according to published criteria^33^. Predictions of snake EDC genes were validated by BLAST searches in the transcriptomes of *P. regius* and *E. coloratus*^36^,^37^.

### Bioinformatic analysis of gene promoters and amino acid sequences encoded by EDC genes

For the assessment of transcription factor binding scores in the promoter sequences of SFTP genes, the JASPAR 2016 server (http://jaspar.genereg.net) was used^58^. Primary and secondary structure analyses of the proteins were performed on the PSIPRED protein structure prediction server^59^ and using the software tools at the ExPASy SIB Bioinformatics Resource Portal^60^.

## Additional Information

**How to cite this article:** Holthaus, K. B. *et al*. Identification and comparative analysis of the epidermal differentiation complex in snakes. *Sci. Rep.* **7**, 45338; doi: 10.1038/srep45338 (2017).

**Publisher's note:** Springer Nature remains neutral with regard to jurisdictional claims in published maps and institutional affiliations.

## Supplementary Material

Supplementary Data
