# Supplementary material for: Identification and comparative analysis of the epidermal differentiation complex in snakes
Source: Sci Rep. 2017 Mar 27;7:45338. doi: 10.1038/srep45338 (PMC5366951; doi:10.1038/srep45338)
Supplement: Supplementary Data [file srep45338-s1.pdf]

Supplementary Data

Identification and comparative analysis of the epidermal differentiation complex in snakes

Karin Brigit Holthaus, Veronika Mlitz, Bettina Strasser, Erwin Tschachler, Lorenzo Alibardi, Leopold Eckhart

Content

Supplementary Figures S1-S10

Supplementary Tables S1-S6

A

>Pb_CRNN
MSHLLRNIHSIICVFEKYAKNDGDCSTLSKGELKQLIQKEFAEVIVDPYDPKTVETVLHLLDTDCDGKVGFEEFTVLLFKVVKACYKKV
QECQVPADGPSQKHNISTQQQEIQLPQVPPCPCQKLLPPKEVLDAEELPQGQEKEKPTLEISCDQPGKEQLQQEEVSAHPRTQTSRVQS
IQKTIEEIIRGNPTCQTSQVEMRTEQGTEQAQEHQRAVPPKNRCLQEPLSPDQEPSQDVLQNVTQQSFQQHQVEERCNTRQKEETSQTL
VQERQTQQSALTMEQIPTGKVIHEPLQETHQGPFGGMNQQVQAEEKRSASGHIRNPQVVQICDQEFNSVTQGQHQIGQQRRQAQGIEQS
SVVQQDQEVQVLGQVISGPQEHQTLTPQVYQSHQEEERKPPTLGQSQYGLSEQRALPQEVIKPQVAERCPHCESPETTVAQGSQESSAR
WPFQTSRSQLRQKPLHFPPPWSTKQ

>Pb_EDCATM
MFFHKKLGWLTPTVVTSPSDDCPALSLPTCSAPCTGACVLPGLGPGSPAACTLLCEAGPNPPVSSPATGGCPACEGLSAPVLKASESPT
MLESCSPPGASTDPWREENMWEGDLPDLSVFLTVPPVPGGDSPCALGRQPDCLPPPCLSHLPLCQDPCSSLCYIPCATMTTNLPPCGVQ
CRNALSRAMGLPLARKCASAIPCTSTCPCATKCANALPCAVEIPPAPNCASTASCATKGATTIPYATKGVTTTCPCATKCVSAMPCAMA
IPAPPQCTCPAPCTTKSAISAPCTTPCPSIIPCTPLCCCKRPPSCVPQPI

>Pb_EDCG
MGCCDCCNSGRSTIIMCMPSHGYQSCYPPGYMQSYGCAPCCCMPIYCTTVRRNRSCCGCGCGCGC

>Pb_EDCM
MSCCCCCGGRGCGCCHRHRPRVVYYVGRPISVRPCCVRGYSYTSCCGHARSCCTPRYRAPYQQYYNQVKKY

>Pb_EDCP
MSFQCKQACPCPSICIKAVPVQAPCSPPSLCPCPCAPVSVKTVCPDPCPAPKGPPCPQKSCPDAKAPAAKCPPPCGTADAKSASPCQSS
DCATPCQATVCVPPCQCQCSACAPCQAQTCIPICGNPCQEQCYAYIPVSISQCSCGCSRILLVPQSSCAKSCAGSCTPVCVAPCPAPSP
PPCTCACSCGPCCS

>Pb_EDCS1
MTCSCCSSCCGSTNRIICVPGHSYYVPPSACCGSYSGSCCGSSCCGSSCCGSCCGSCCSSPPSNIVYTSPMQYCCPPMKQCPPSLQCKQ
SGYPSKKCC

>Pb_EDCS2
MTCGCGSSGSDSPTIICIPCGGQPCCSPCCCCPCYGGMQSCCCMPCCAPCGYGGCGGCCGITAYRLPSRRRSSCCGCGY

>Pb_EDCS3
MTCGGGCGSCGDSCGTCGAMLLCVPCCGCQACCSPCCSQSSCSPCSCSKCSCGQSSCSPCSCSKCSCGQSSCSPCSSSKSSCGQSSCSP
CSSSKCSCGQSSCSPCSCSKSSCGQSSCSPCSSSKSSCGQSCCSPCSCSQSSCAQSCCSPCSSSQSSCSLPCSQSCCPITCPPCCSK

>Pb_EDEPK
MSTEQQQRKQTSVLPPGLSELTTESTPEEAPQKEHDRESLSSSEQGNALPEDPKESHERKELSSEFSAIEMEPGASSEGTSGAKHGPLA
IKEEEHLPLVDQHCREPPTLPLAPSKETLKPGQPLLPVREAEGHPATQPPALGGQKQQQQQQK

>Pb_EDEPT
MAYQYKQPCPPPPACGVQCSAEADDACTLTGVAPCDPTCDASPSGACNPPDTLLYQTRQPCEATPCGPPPCPTAAPSPCALPCTSLYTV
TCAEAPSTPCGSATWQPCTPLPADAPPCPLEEACTRGPGCPETCSDPQGDMGMPYVKPGSTVCLNPCGAVSVKPSSPEEEENSIFLASY
TPHFT

>Pb_EDETM
MACPGELCSYNTSYSTSLPSSSFLLQPPSYMAETRLTGLEVLVPESTSCEIPDGCLHPCGSPAFCCHEPPVVLAFFETSLYEV

>Pb_EDGPC
MGNMESDSGGGCGCNCNCGGGSSDSHVICVPSGGGSGCCCPPCCSPCCCSPCCCSPCCCSPCCCRPCCSPCCGQSSCCAPCCKQCLK

>Pb_EDGY1
MIFYTQADSYEPYLPYYLSYCPPCYPPCYPQYGYSSGFGSYGQGDSYNYGYDSGSSSIPSIPPCSYGYRYTRRGSCYEPCAYRLGYGAR
RNFPSRRYYNPNLW

>Pb_EDGY2
MSYYDPCRSKCYSSGSSLGGYKRRIRYSTGSCYGGYGQGTRYIPSCGYGYGGYRGQTYGRICYQPLDYGYGYGYGNKYCPPQWYGGYGS
GGYDYGCRYGGLYGRRQSICYDPCYGPRYQYGSRRGSFCGPW

>Pb_EDHEM
MGCTSPNTGNEEITMMRYIRIEPLEELGLRSCPHPKHHEMMDLFCAPDLEILLEEPDLLELISIEDTSPHPEYYRRTRIYDMIELPLSA
GAGKDSGCSSHKNADPGAEHSQEDSEKATSPSSCEPKNGDTNNLPASRNPSDPATVIYSFTVEYQCSPLLKYCTLEEMGYRPIQLM

>Pb_EDKM
MSNLFRAFTEMIEGNSKVVWKKAHDSEMLKKSEFKKLIQQELSPSKTHKYKQVKHLHDSDTELMNEKELTSQLH

>Pb_EDP3
MSQQQQCKQIPCTPPTSCKCTSSCQKPCEPLACQGSAGPQCPGSTGNSSQKPTCPPGKNECFCHCHQQQQC

>Pb_EDPAM
MTHQCKLPPELPPACLKLATKTSLQTKSPPPQTVTVCAVPTPAPSPPPPQSVTCCGVPLQVPQSPPPQRVTVCAVPAPAPSPPPPQSVT
CCGVPLQVPQSPPPQRVTVCAVPAPAPSPPPPQSVTCCGVPLQVPQSPPPQRVTVCAVPAPAPSPPPPQSVTCCGVPLQVPQSPPPQRV
TVCAVPAPAPSPPPPQSVTCCGVPLQVPQSPPPQRVTVCAVPAPAPSPPPPQSVTCCGVPLQVPQSPPPQRVTVCAVPAPAPSPPPPQS
VTCCGVPLQIPKTPPPQTVTICAIPTPALAPPPAQSITCCGIPLQIPNPPQTVTICASPAPASAPAPTLVSVPCSSACTSCVPVTLSLV
QPTCVAVPQCSSCCKTCHGS

>Pb_EDPCCC1
MAHCSKCGCSPCCCKKHGHSHCRKKCRCNKCGCSPCCCKKHGHSHCHKKCRCKKCGCSPCCCHLCSCSPSCCQQCGCSPCCCQQCGCSP
SCSQQCGCSSSCCKKCGCSPCCCQQCGCSPSCCKKCGCSPCCCQQCSCSQCCCEKSCCCTPCSCCCSPCCGSKGCSAKGCGSTGCFPQQ
CEC

>Pb_EDPCCC2
MTCCSKCGCSPCCCKQSCCSPCCCNQSCCSPCCCKQSCCSPCCCKQSCCSPCCCKQSCCSPCCGQQSCCSPCCGQQSCCSQCSCGKSCC
CTPCSCCCSKGCSSKDCCSKGCSSKGCSSKGCCSKGSCSKGCCSNQCKC

>Pb_EDPCCC3_partial
MASSGTKTPKCSKCGCSPCCCPKASSSTKCSKCGSSPCCCSKASSSSKTKCSKCGCSPCCCSKSLCSKCRCSPCCCPQSRCSKCGCSPC
CCPQSQCSKCGCSPCCCPQSXXXCSPCCCAQPRCSKCGCSPCCCPQSRCSKCGCSPCRCSQSRCSKCGCSPCCCPQSRCSKCGCSPCRC
SQSRCSKCGCSPCCCPQSPCSKCGCSPCCCSQSRCSKCGCSPCRCSQSRCSKCGCSPCCCPQSPCSKCGCSPCCCSQSRCSRCGCSPCC
RSQSPCSRCRCSPCCCSQSRCSRCGRSPCCSQSCCSRCGCSPCCRSQSPCSRCRCSPCCCSQSRCSRCGRSPCCSQSCCSRCGCSPCCR
SQSPCSRCRCSPCCCSQSRCSRCGRSPCCSQSCCSRCGCSPCCRSQSPCSRCRCSPCCCSQSRCSRCGRSPCCSQSCCSRWGRSPCCCP
QSPCSKCGCSPCCCSQSQCSKCGCSPCCCPQSPCSKCGCSPCCCSQSRCSRCGCSPCRCSQSRCSKCRCSPCCSQSCCSRCGCSPCCCS
QSRCSRCRCSPCCCSQSRCSRSGSSPCCYSQSRCSRCGCSPCCCSQSRCSKCGCSPCSCCSQSRCCSKCGCFPCRCSQSRCCRCGCCPC
CCTPSCCPRCNCPYCQQNQSCCCPCCCRYSCCCPCCYCSCCNSSPLKCQPK

>Pb_EDPCCC4
MTCCPICGSSPCGCAPCYSCSPCCGCGSCGSPCCSSCGSPCCSSSCSAPCCSCSPCSGCGSCGSSCSCPQCSSSPPCCACGSCCNRQSS
GCGCGCGCSPCSSCSPCCGCGSCCSPSCSSCCGSPCCSSCGSSCCGSGCCFPKQCFS

>Pb_EDPCS
MKPLPPCGWFCFCNVFLQRLGLHDPCKKVVDPFQLHLSLFLLLPSLCTMEFEYVFPELLDETDPTFLETYRQRPWKWVDPCKPRYCPPK
YFPPCPPPCPPPCPPPKPQRPIPCCPLYPLPQCPKYFPPCCPPYPCCYPPQWCYPRPLPCVPQKPWYPCKVPCPPGWVPKYPRPYDLLE
LY

>Pb_EDPKC
MASSHNQHQCKQAPTLPSELCKSAPCPPDQQCKQPPVVVVPTPCPEPKPCPPPEPPCKEPPIVIVPFPEPKPCPPQEPLCKEPPAPTPC
PPKEPEPCPPKQPTPCSQQKSQCKEPPVVVIPTPCPEPKPCPPQEPPCKEPPVVVIPTPCPKPKPCPPQDPPCKEPPAPTPCPPKEPEP
CPPTPCSQQKSQCKQPPVVVIPTPCPEPKPCPPQEPPCKEPPVVVIPTPCPKPKPCPPQEPPCKEPPVVVIPTPCPEPKPCPPQEPPCK
QPPPCDQQQKKQPCSWPPQNK

>Pb_EDPQ1
MYTKGYGDGGGSYRNYGRSACCEPSCHSVRRRSEVKCCDTSPQSCCPPVQKCCLPRQKYCPPVQKCCPPKRKYCPPVQDYCPPVQPCCS
PVQKYCPPVEPCYPPVQKYCPPVQPCCPPVQKYCPPVQPCCPPIKKYCPPIQPCCPPAQKYCPPVQRCYPPPQRYCPPVQPCCPAPQRY
CPPVQPCCPPPQRYCPPVQQCSPPPQRYCPPVQPCCPPPQRYCPPIQPCCPPQQRYCPPVEPSCPPLEISQVQQVCRVPPHLLK

>Pb_EDPQ2
MSYHQQQCKQPCQPPPQCQKTSCPPACPPQKCVPKPCPPQECVPKPCPPQECVPKPCPPQECPPKPCPPPQQCCQNPKRC

>Pb_EDPQ3
MSYLNQQCKQPCPIPPQCKKTIPPPEQCCPPPQQYCPPPQQCCPQQQDQDPKCC

>Pb_EDPS1
MFHCCVPSCSMGPTAPLCFQPCQQSRSMPTISIPCRPVHPSMPPTSIPINLCPVPSMPPPSSSPSICIPLCPSPSPSSQRQPSIPLCLS
PQPSGSSSGSNSRISISLSPSPSSSSQSQLSIPLCLCPQPSSSSSGSSSSPSISISFSPNQSSSSQPLYCIPLSPSQPPPSISLSPSQS
QSSSSHSICIPFCPSPLPSSSSQPSFCIPLRPSPQTSSSSSSSSPRISISLSPSPSPSGQSQSCIPLCFSPQPTGSGSGSGSSPRISIS
LSPSPFSSSQSQPSYCIPLCPSQPPPSMSPRPAPPPTCCIPIIIPSNPCFCGC

>Pb_EDPS2
XXXSSSGQSQPSIPLCFSPQPSGSGSGSGSGSSPSISISLGPSHSSSGQSQPSIPLCFSPQPSGSGSGSGSGSSPSISISLGPSSSS
SSQSQPFYCIPLCPTQPSPSMSPSSSSQPTCCIPISIPSNPCFCVC

>Pb_EDPS3
MSSCCVPSCRVGPIAPLRVQPRDQSSSLPSCISCRPVQPSMPPTSIPISLSPVPSMSQPGPSPLSSCIPLCPMQSPPSMSLSPSPPPPT
SSQPSYCIPLCPSQPPPSMSPTPSPPPSCCIPITIPSTPCFIVF

>Pb_EDPSQ
MDCCSQQCKQPCLPPPICQQKIGIKRTLWQQRGSSQCTKPCSPSWPQSYDPECPPAYPPQGRAVCWAQPVQQCPSWPRQPCSPPCKSVS
FNSKGLRAPHGMQQSASKGKRQCLLQCSQDGPHQCLKVCAVKGCKKCANKGSPQCVTKESRGGLPCAYQPHTVKGDWTWFIKGNQQCVS
QPCVTKVNPLYSSKGSQQYRRKGGYCKTQLKVSSNGQKYCSASNNWPW

>Pb_EDQL_partial
MCSHHDRGCHGVSHDHGCHSGSSCHEDHGSCHNRSSCHDGGNPSFRVMTIPVAQDKNPMPIVQQQSPVVYPAPKLQQTQQLKQPTPYPP
QQQLQQPQQQQKQKSKTQRKSCXXX

>Pb_EDQM
MCSREDKDQCYKQEKDESPCCYSHRSSGCGNKPSSRACSGSYGWGPSSPYCQGKSDQPQQQQQKKQGCQLPPQK

>Pb_EDQSG
MQQCKQPQGFPPVGQMSTAAQCKASAVSQSQGATVDQCHGSSGNQTQGSTGIQCGGSAVSQGKGSTGNQCQGSMPSQTQSSGVSQWRGS
TGDQCGGSAVSQSKGSTGNQCQGSMPNQSQSSGVSQWRGSTGDQCGVSVVSQGQESVAFQDQESMVFQGQGSPRNQCGGPSVSQDQGST
GCQCHESAGTKY

>Pb_EDSC1
MSQQIQQGSSCCCCCGSSGGCCSRGSGGGCCGSSGGVVYVQQSQQSSRGCCINRTGACCGRCCGRRCGGGCCRRGSGGGCCSRGTGGGC
CGSSGGAIYVQQSSRCCCINRTGPCCGRCCGRRCGGGCCSGGSSQQKLK

>Pb_EDSC2_partial
MSQQQQRGGCCCCRCCGGGGGGGCGCHSGGGSQVSRSSGGGCCGRSSGGSSQQSXXXSSGGGCCGRSSGGSSQQSHSSGGGCCGRSSGG
SSQQTHSSGRCCGGGGSSQQKMK

>Pb_EDSCP
MVFSCHRPTCTSPSGPIFPISGGSHSCVPVCQPTCSIPSGPTCTRPSGPIFPISEGSHSCVPVCPPPCCIPCGPPTVCVQPPPPPCVPV
CPSPCCIPCGPPTVCVQRPQPSCIPVCPSPCSNPCGPPTVCVQPRSSPCCIPSCTTPSRPVITVSPCCSVPSCSIAPLCPQPGSPSCVV
PSGPILPVSSGQPSCIPISVHPCSPCLTFF

>Pb_EDSPR1
MACPYQQCKQPCLPPPICGKGGVKQCAVCPPACPCQSVCAVPSVKVVGDPCTPICIPPCAPKCAVDNICVQSCPPSGQSICVQPQVVCP
CDSKCPDPCCKQGSC

>Pb_EDSPR2
MSQCKQACKAPPCPEPCCPTKCPPKTCCSSPGTKAVSKKGPCCPPSADVKCPPPKVKEPSKDCCC

>Pb_EDSQ
MSYQCKQPCLPPPFMKGTTVCAGPGTTVCVSPTQGQSIEVCASPTGAICVTQGQNQAQCANVCQDCCGSVVVSPVQNQGFSPCATLCTK
CNGAVCVTKAPEQSSAVCVPQQQACQCSVCQGNISVAPIQSQSGACATICASPCSSVCVTPIATPLGPAATICTSPGACATVCQDPCGS
VCVTSKGATKCATKCATKCVAPGATVCVDPCGGVKSVKSCTTKCTSPCFAVSNEQVCVKACPSISMNQCNVKKC

>Pb_EDSRWM
MNFYMPQEYWDLNSWETNYENNYDFPSEYYYAYNPRSYILDYQSRWGSPSYSSCSDCCDPCYSAICGSPRYRSRSSYCPSCAPSSNSSW
GSPDCSSNSSCCPPCWTCGSCNPRYSPICRPQCHRSRSVSPFRSNSRSSCCPPCCRCGRWAHPSSSSSQSPDGSSNSSYCPPCWTCGSC
NPHYSPICRPRCHRSRSVSPFRSNSGSSYCPPCCRCGHWAPPSSSTSQSPDGSSNSSYYPPCWTCGSCNPHYSPICRPRRHRSRSVSPF
RSNSRSSCCPPCCRCGRWAHPSSSSSQSPDGSSNSSCYPPCCRCGGWAPPSSSCSQSPDGSSNSSYCPPCWTCGSCNPRYSPICRPRCH
RSRSVSPFRSNSGSSCYPPCCRCGGWAPPSSSCSQSPDGSSNSSYCPPCWTCGSCNPRYSPICRPRCHRSRSVSPFRSNSGSSYYPPCC
RCGGWAPPSSSCSQSPDGSSNSSYCPPCWTCGSCNPRYSPICRPRCHRSRSVSPFRSNSGSSCCPPCCRCGRWAPPSDSCSQSPDCSCH
SSCCPPCCTCGSCNPCYCPICGPQPSNFRSSSRRCPCCCRCGRCAPPCCCSSQCCRSSCSCCRPCWTCSSCNPCYCPSCRPQPPNSSFS
SCCCPPCCPCGCYGPCYDPSC

>Pb_EDWM
MSEERIYSSGREPYFNLNSTWYDPAGSWLDNRRKPFCYVENTACVTCCNPRTNVPRRGGHNYRCYCYRRSTCRPGGNPRVRCCVHNPSG
GPRDYWGRPIGDACNGCTGGYYSHGDCGSCCGSSGECGTGGGLVACAQPCVSSGGVCAEPGCRPAGRGVCAEPCVMSSGGWGSGRGVCA
EPECSASGCGSRRRGVCSEPCARASGGC

>Pb_EDY1
MIGITILSSHPSEGTKAIYCPPTIYKPSGSDSSAYPPYTSLAGTAARCYIPRKSRYLPGPTYYLSGWDCSRPMPHTYRSTYGPCSYEYL
LDNSQGYYDPCVYKYLSKGVQRSYDPYGLEYLYSYRRRYYDPSDYRYYPSYGGRCDPCVYNNPPSYGRSSYSSRGYTYRCGFSNDPCGY
SCAPGYRQPRYADRRRYSSRPISSDPYGSCPESPYASECRRPSSDSSSYCGPC

>Pb_EDYM2
MPYYAYHCKPIGPPLPLFVKKRLPRYGSQYFPLYGSRYVSSRSIPFHRRGIWMTPYSLASVAGYQPWIIPRVSEACSSKSPKLRTTRNP
VRPYVDSYQYPLQTTVPYDGMTKGPHGPINKGSLPCVIPERVLRVTKGPVLYSSKDLHPSSAQIPLRRACKGPVFYASQDPLLCSSKEE
LPRVSKGPDQYPLKGAQRSRAADSYLPMTEDPPQSGAKVTQPRATKGPRLIATKVPRFSALTVSRSFLKRGVHSFLTKTSQTNLCKGSR
SSLSKTSWLSLGKGSQPSLVRTATTPAPKTLSKNVKISSTGKKYCSTTKWPF

>Pb_LOR1_partial
MTSWQQTSGCCCSSGGSICCSYPCQGESSCCCGGGRGRDGRGSPNQQSQGSICCSGGSGGSSGQTIIVVPGSSGGDGWCCCCGGGGSSD
GSSGGSGQIIISSGGGVGRSGDRQSSECCIGGGSWGGSGSAMSQQKCDGGEICCGGVSGGGGSPGASGGVTINMGGGSGGGSSGCQTII
VPGGRRGSGSGQSLGSSMGGGSGGGSSGCQTIIVPGGRRGSGSGQSLGSSMGGGSGGGSSGCQTIIVPCGRRGSGSGQSSGSSMGGGSD
GGSSGGQTIIVPCGRRGSGSGQRSXXXSGHSSGSSMGGGSGGGSSGCQTIIVPCGRRGGGSGQSSGSSMGGGSGGGSSGCQTIIVPGGR
RGSGSGQSSGSSMGGGSGGGSSGCQTIIVPGGRRGSGSGQSSGSSMGGGSGGSSSGGQTIIVPGGRRGSGSGQSSGSSTGGGSSGGSSG
GGKTIIVQCGGASGGGSSRAQSSGCCIRGGMGAGSMQTKQPISIPPCIGPTK

>Pb_LOR2_partial
MSKQIQSSVCCGGGGSSAGCCVRSRSGGSSCCCACCCCCRGGSGHVIAVSGSQGSSCCGGIQQISSSSCCGGSSTGVVVVPGGQSSGCC
IGGGHGGGICQQKIPLIGVGGGGICCGGGSGGGSGGQTVIVPGGGSGCCGGSGGVRVVGGGSGSGWCCGGGSGGQTVIVPGSGSGCCGG
SGGVKVIGGGSGSGWCCGGGSGGQTVIVPGGGSGWWGGSXXXGGGSGGVRDVGGGSG

>Pb_PGLYRP3
MVTLQILFLILCSLSQATGCFQLITPSKWGAKPANCSQPLRDVPPEYVVIIHTAGNPCRTHADCRKEVKNIQDFHMNLKGWCDIAYSFL
IGEDGYVYEGRGWRNEGSHTYGYNDLSLGIAFIGTFVERSPQDVAWKALRCFLNFSVKIGYLAPEYILLAHSDVSDLVSPGEFVRAEIS
KWPNYKHSFYVLDRGGQ

>Pb_SCFN1
MTYFLDSVCTIVGIFHKYAQWQGRNLTLNRREMKMLIQTEFAEVLENPCDPQRVEFTFQLLDVNGDSLVDFNEYLIFIFQIAKACYSYL
QPREYLLQEDGSRALHEGEQGGSKRDHWQLQDEEREEDYVHERRGSDRTPLRPMEDSRRGELGKCYLPSEPEEEEEEKEEERNFQGRDR
ELRDGDRRIHPSWEHQERETERRRLEPKQQEDIETPEHHQLRQREPALEEGSPRQSRRRRNDNDRRQADEHLDRDEHRGRETEHREAEG
RLFRCGSKARVDQGNHHAEYNSERSQASCEPRELEDGRRDLETHDELRTRAFEPHVTDERRDQIRNRRPVDDYEEHRPLPREGERRSQS
HETEHRESERRRRDHSAFQSRDDVRIANRAEEEEVRGCERRRDEDEWSRPRQCEALRRPEVEELEHRERERRSYSPELENMDRRNQPHE
SEGRLRRSRHREDEERRRQQFSWEETQATESRRRRPRSHEPEPRDPKYGRSQRYEEEPKRADQRRQHNYEVQSPERDVARRRRLQTRES
AEREDNQERQQYYERESRGSEGDRRRPQLCYSEPREGEKRRHQLTDSRVVERERLQSRESVTRPTQRDRFSPSDSSGEYDWSRQQIYES
RDIEQREGRRRSYDRRSCGQRQIQGIDIDPREGEGQRRTNAEAGDVDQRQTQTRGVDHGESEQQRRTSSRDSGDKDIDQRRTQTDEASS
RDGEDQRRTSSHDSNTRQVNQKMMQTSDPDFRDTEIPQHNKGNAPQPREVPSLSNQTGDREFAEDLLRDQRPPESGESQLSRKPRQHKA
EQSQSIPRTLEQKQATRGSQCLSGPELRGPQRRWLQPHQTEAQEGEAGVEQTQLKVGSPESKRAASQPGEQQTTEGQESRVQRHHPKPA
TQDEGESVAGKGGKAQEPATVKRQQLQAQGASYEGEGPQPEVEGPKVEEDDENGPRAQESQPPLEEDDQQAVTEKPCDSLGESKSSVVC
NPLYEYLLAQKKQEQP

>Pb_SCFN2_partial
MACLVDSVCTIIGVFHKYAERRNACSTMKRREMKRLIQKEFGEILENPRDPQIVKLTFQLLDVNRDSLVDFNEFLLLIFEVATACYSSG
YPKECLSPKEERSRVVRNGEPRGNENNHRQFQDEEREEDYVHERRGSDRTPLRPMEDSRQGELGKCYLPSEPEEEEEKEEEERNFQGRD
RELRDGDRRVHLSREQHKKETERRRLEPKQQEDIETPERHQLRQREPALEERSPRPSREPMRRNDSNRRAGDPLDRDEQDLYSPVVTSR
REDGRQRRGRETEHREAEGRLFRCGSKARVEEGNHRAECDSERSRASREPRELEDGRRDLETHDELRTRAFEPRVTDEXXXATAEARRP
RHHEDDEQRRQQSNREETRATESRSRRPRSHEPEPRDPKYGRSQGYEERRRDDPRRQRNYKVESPERDVARRRRLQTRESAEREDNQDR
QDYEHESRDSEGDRRRPMILEPETRDEDQRRPQETGSRYGERERRQERDGRIRPTGRELGSPSDGGQRELDSSRPQISRCRTAELSRSE
PRSRKPARRG

B

>Pb_Beta1
MSGSGVKCVTTPCITSCPDAKVVVHPPPLILTLPGLSLRTSPNQCLLETQTSCLTNGSEVGCNEGSTAIVTKSSGVCALSGGDTPCCTT
TCPDSQVVIQPPPVCITIPGAVLTSYPNECLIKTSTPCVPSGPQPPALTRSTSVSDCSLTPRRLSRSASVPSGLDSLSRCVTRGPTTKV
VIHPPPIEVTIPGPVLEIAAQECAVEVYNPCSSNNALTSSDQKAITSSDEGEVKALTTQAKSCTTVCGLMDTSSCISQGPEMKIIIQPP
PIEVELPGPILEVFPEACKIETLNPSPLEPEAITGSETKALCSSKMTSTALATTSNVKRPLPNLRRAPRPWAEMYSRSMTPRTIALSQQ
TRLAKYRNTLHSMNFQPSF

>Pb_Beta2
MERRPLCYASCPPSTVTIQPPPFTLTIPGPAIFCPNQPFHIMQYNPCARDGMGIGGRMMFSDFISEDLPDLKLESSSGHGSALVTLYRS
RMALLLAMTGSN

>Pb_Beta3
MSECYASCPASTVTVQPPPFVLTIPGPALYCPDQVFGIEQYNPCAGFGGMVTRGGGGIGVEGRGGTLGALGAGRGGWGGSWSGGSGIGS
RIGGGLGIGGVGGPRGGGLGIGGVGGRRGGGLGIGGVGGPRGGGLGIGGVGGPRGGGLGIDGVGGPTGGGLDIDGPRGTTGSWSGGSGV
GGTVGGRRGSIGAGSITGGDLGTGGVGGTRGGCLSTGGTRVTTGGWSGGSRTGGSVGGRRGSIGAGGTVAGWSGGGGRRGSVGTVSVTG
GDYGLSGAGGGWSSSHRISGSHRWSGSGYGGSGGYSGGGYGGGYGSSGYGGGFSSGRYGTSGYGGGYGHTGGYRRIKYSVGGGSGHRSG
GFSSRSYGGSYGGTLAVLPGPSDISCY

>Pb_Beta4
MSTSYNPADVCAFSCPPTMVTIQPPPFTLTIPGPTIHCPDQPLQFQQHNPCVFNKEHSPLLASRNSSFYSRALPSGSYTSSIYRY

>Pb_Beta5
MSANRSFWPNCYAQCPASTVTIQPPPFVLTIPGPALYCPDQPLGIDQYNPCEYGAYPLPLGRGGSNLTNFYSRGLPSGSFSSTTYRSSN

>Pb_Beta6
MASGWNPCCASCPPLTVTIQPPPYTVNIPGPSLYCADQPLCIEQCNPCVPIQPISHGGYSLLSSSAVSDFSSSSQKSLRSQRF

>Pb_Beta7
MASGWDQCYSNCPPMTVTIQPPPFTLNLPGPALHCPEQPFCIEQCNPCVPTPHCDNSLFSSTASGFSSSSVASQKSVSGRQSSGCFPCR
RC

>Pb_Beta8

MSFGSNSCYASCPPVTVTVQPPPFTLNIPGPSLYCPDQPLCIDQYNPCVPPPICPPRRSYTSNSISSSSSLPQKSLPRSQSEWSSVSQR
F

>Pb_Beta9
MFCGWNPCSSSRPMTAQPPFFSLNIPGISICCPEWPCCPEQYNPCAPVQPCGRMNYGRYGSYWSGRASDCNSLKFSAPKHLHQSQTKSL
HY

>Pb_Beta10
MASRWDLCSFNCPPTSVTIQPPPFTVSIPGPSLYCPDQPLCIEQCNPCVPCYGGSSYGRSRPVILASISSQKTVSQYQKGSEPFKRF

>Pb_Beta11
MSFGWNQCYPNCTPMTVTLQPPPFTMNIPGTSHCCPDQLLCVDSCSPCSPTVCAPMVCSPTVCSPTLCAPTHCGPTHCAPSQCAPVVCT
SGLNRSRYSSCFSSCSLPSRKCLPRVQSVCDPCTKY

>Pb_Beta12
MASRWDLCSFNCPATTVTIQPPPFTINIPGPSLYCPDQPLCIEQCNPCVPCYGGSNYGRFRPSSAISAASIYSQKSVSQQQSGCDPCQR
F

>Pb_Beta13
MNAARVYSGWDPRFSNCSPMTVTVHPPPLTLSIVSPAHYSPNHSPCIEPCNPCAPFQCWDGRNRYGYHALTNGVAPCLTSTNSSSQKCL
PRSKSGCVPCRSY

>Pb_Beta14
MTSYKLCTCVSCSSKLPYSKAKPSCLCMFVSEINIPSHQELVPARSETLSEAQNIRRFSRQKKSNLTCLPTSQLLGHLGRGPTRRISYN
LQRKPPLRSYRYIKC

>Pb_Beta15
MAFWDYPSFNSGLCGVSVSCVSQIPASEVIIQPPPVALTIPGPILSASTEPVAVTQNSPCALGALGACAPPGYGYGSGRRYHYGICARP
SYAQKSS

>Pb_Beta16
MSYCGPVCHVSCASSGGSCGLVPAYGSSSLSGAVSSYYGGGPITCSSQLTGSEVILQPPASVVTIPGPILSATTEPVTVGQVTPCSYSH
PLSFVGGSSYGGYGRLGYSGYGSYGTWRRYSRRCLPNYCGPC

>Pb_Beta17
MAFCGPPCPLPPCPIPSCAIPSCASCPSVGFGPGGLGGLASRSFGLPSGQPASSLGTLEGVTPSCINQIPAAEVVIQPPPVIVTLPGPI
LSASCDPVAVGGNTPCAAGGYGQGLPTGLLGGGSRQGSRYRFVGNRGSICYIPC

>Pb_Beta18
MAFCGPPCPLPPCAIPSCASSPSVGFGPGGLGGLASRSFGLPSGQPASSLGTLESVTPSCINQIPAAAVVSXXXEVVIQPPSVIVTLPG
PILSASCDPVTVGGNTPCAAGGYGQGLPTGLLGGGSSGPRPGRRFTFVGRRGSICYSPF

>Pb_Beta19
MPHCGPSCAIPSCASSPVVGFGSAGLGYRGLGLGCGYGLGYGYGLGSGYGLGSGYGLGSGYGPGALATSSGSLGTLAGVIPSSINQIPP
AEVVIQPPASIVTIPGPILAASCEPVCVGGNTPCAAGGFGRYGGYLGGRWGRLGRRGSVCPLPCPLPC

>Pb_Beta20_partial
MPYCGPSCAIPSCASAPVVGLGSTGCGPSGLGYRGLGLGYGYGGWGESASNLGTLAGVNPSCISQIPPSEVVIQPPPVVVTVPGPILSA
SCDPVSVGGYTPXXX

>Pb_Beta21
MPYCGPSCAVPSCASSPVVGFGSAGLGLGSGYGLDSGYGLGSGYGLGYGYGSGALATSSGSLGTLAGVNPSCINQIPPAEVVIQPPASV
VTIPGAILSASCEPVCVGGNTPCAVSDSGLRGSWGYGDWGYGGLGLRNRGLLGRRFPLSRRGSICYSRRGSICF

>Pb_Beta22_partial
XXXPGPILSASCDPVSVGGYTPYATGGFGRYGGSLGGRLGRFGRRGSVCPLPCPLPCPLPCPLPCTLPC

>Pb_Beta23_partial
XXXVPGPILSASCDPVSVGGYTPCATGGFGRYGGSLGGRLGRFGRRGSVCTLPCPLPCPLPCTLPC

>Pb_Beta24
MSCCVPSCTVPTCVPSGSPPICYPVGGLGSLNPCAISTAGGVSASSLGIVPGASVGCINQTPPSELVIQPPPITVVIPGPVLSASCEPV
RVGGFTACSGGSSNGGSSRVRCYPCNPCKV

>Pb_Beta25
MPHCGPSCAIPSCASSPVVGFGSAGLGYRGLGLGSGYGLGSGYGSGALATSSGSLGTLAGVIPSCINQIPPAEVVIQPPASIVTIPGPI
LSASCEPVYVGGNTPCAAGGFGRYGGYLGGRWGRLGRRGSVCTLPCPLPC

>Pb_Beta26_partial
MAFCAPVCGLPTCAIPSCASAPQYGLGSSGGSGGLGLGLGGRGGLGGDSLGGSTTSGQLGTLAGVNPQPINQIPSAEVVIRPPPVVVTI
PGPILSXXX

>Pb_Beta27_partial
XXXGRGGLGGGSLGGSTTSGQLGTLAGVNPQPINQIPSAEVVIQPPPVVVTIPGPILSASCEPVAIGGNTPCALSGSGPLGRPLALGTG
PFGRCLLGGRGNPCLPPCGF

>Pb_Beta28
MSYCGPACAIPSLASNPIVGFGSAGLGGPGYGLLPYSSGSSALAESSGGLGTLAGITPSCVNQIPPAEVVIQPPASIMTIPGPILSASC
EPVAVGGNTPCAVSGSGSDLFGNCLPGNPGLGLRRGTLLGRRSLLGNHGDVCL

>Pb_Beta29_partial
XXXCAISTAGGVSASSLGIVPGASVGCINQTPPSEVVIQPSPFLITIPGPLLSASCEPVRVGGFTAGGGGSSNGGSSRGRCYPCIPCNP
CRS

>Pb_Beta30
MSCYVPTCTIPTCVPTCPPASSSPICYPVGGVGSLSSSSSISSGGGTAASSLGIAPGATVSCINQTPASEIVIQPASVILTVPGPILSA
SCEPVRVGGYTACAGSSGRSSRSMMSGSRPYICRS

>Pb_Beta31
MSCCIPACLPPCPPPCPPTCAVPSCAAAPTIGLGSCGSGSGILGYGGGGAASASSLGILPGASVGCLSQIPPSEVVIQPPPFVITIPGA
ILSASCDPVAVGGYSPCASGSGGYLGGGLGGICRPRRKFSICKYPC

>Pb_Beta32
MSCCRPCCPPTCAIPSCASRPLIGLGGCGGGSALGGFGSGGGGAASASSLGMLAGVNPSSISQIPPSEVVIQPPPVVVTIPGPILSASC
DPVAVGGYSACSSGSYGSYSRGLLGASSRGICAPRRRYSICSSPC

>Pb_Beta33
MYCGSPCPPTCAIPSCRPTLGLGPCGLGSGSGGIGGGGSGIIGYGGGSGLAASASSLGMIPGVPVSCVSQIPPSEVVIQPPPYVLTIPG
PILSSSCEPLAVGGYSPCATGGYGASGGYGYSRYGLGSSGGYLGSGRCYGSKRRQSVCGYPC

>Pb_Beta34
MACFIPTYTTPAAASNVGLGSCRIGPNISDCGGYGYGGSGGVITGGPSSILGLTSGANIARTSRLPPSEIVIQPPLCVLTVPEPVVSAI
TPPLILGGSSSYIYGFPGYGYGPGSVGSCLRGDIMGGFQNIGQSRGRCNIYQYPS

>Pb_Beta35
MSLCNYCCNPVYRVDTPCILQIPLSEVAVQALPFLITVPGPVMSASPELVAVRGNTPRAAADCYEPYGSGGWVLEGGHGAPLAPIHIPR
GPTKGYF

Suppl. Fig. S1. Amino acid sequences of proteins encoded by EDC genes of the python (*P. bivittatus*). **(A)** Amino acid sequences of EDC proteins other than corneous beta proteins (CBP). **(B)** Amino acid sequences of CBPs, also known as beta-keratins. Amino acid residues K and Q (potential transglutamination sites), C (potential disulfide bonding sites), P, G and S are highlighted by specific colors corresponding to those in Figure 4. Stretches of X´s indicate unknown numbers of amino acid residues, that could not be predicted because of gaps in the corresponding gene sequences. Pb, *P. bivittatus*.

A

>Oh_CRNN
MSHLLRNIRSIICIFEKYAKDDGDCDTLSKGELKQLIQTEFADVIVNPHDPKTIETVLHLLDTDCDGKVGFEEFTVLVFKVVKACYKKV
QECQGTAGGPSKRGITSTQQQEIPPQQISRDGQSQRDITSIQQQEIPPQQISRDEPSQRDISRTQQQDIPPQQLSRDGPSKRDITSTQQ
QDIPPQQISRDGPSQRDTSRTQQRNIPPQQLSRDGPSKRDITRTQQQDIPPQQVSRDEPSQRDTSSTQQQEIPPQQISXDGPSQRDTSS
TQQRDIPPQQVSRDGPSQRDTSSTQQRETQTSQVSPRPCQKLVPPKKILDAEELPQEQERERTRLETSYDEKEQSQQEEASIRPRTQTS
RVLSVQRTIEDMIRGNLTCQMSLAEMRTEQAPAQGHPSALPPRRRCLQEPLSPDQEASQDVLQSVPQQSSQQRQVEERSSTCQEEVTSQ
TSGQDRRSQQSAVEMEQISSGESLRETRQRSSGGRNQQVQVEERSASGHLRIPQVVQICDQESRSVTLRQHQNDQQRCQSQGVEQSSVV
QEDQEVQAPGQVISRRQEPQMPVYRPHQEKERYPTTLECQRGPSEQRAVSQEVTRPQVAERYPRGEPGELTIVQFSQDSSTRRPFQPSQ
FQLKQRPLQFPPPWSTKQ

>Oh_EDCATM
MFFHKKLGWLTPNTVSSPTDSCLSPSTPSCSGGSPCLGSRTPPVHKVFRMAGLKPPASVTSSPLACEGLSSSIPKASGTPIVLMSCSPR
DAGFNPWNRNESAIWKERNIFTNELPNLSVFLSPLPASGGPVPLTLQCQPDCITPLCLTSFPLGHGPCGPLCVVPCATQPVNLFPCGTQ
VQLAPNCANMVPCTVKIAALGPCAMKGASLCNTEPPLASKCASPLSCTMTCPYAAKDANSLPCTMEIILASNCASPVSCATKGAAEMQY
DAKGAALTSPCGSKGADIHPCDVEILPAPNYASPTSCATKGATIIPYATKGATLTSPCGTKGANFHPCGVEILPAPKCTSPVSCATKGM
TTIPYATKGATMSCPCATKCVSAIPTLPQCACPVPCATKGAISAPLTPSWPVLTLCTPNSCHNAPAFTVPQVVQK

>Oh_EDCG
MGMWDCCSCCGNSSGRSTIVLCMPSGGYQSCYPGCMPSCGCAPMCCMPIYCTNMRRNSCCGCGCGC

>Oh_EDCM
MSCCRCCGGRGCCCCNRYRPRTVYYAGRSATAGCCCRPRGCCCGRPGCCCTSYYPSYPYYRVPVQTYGTQVKKC

>Oh_EDCP
MSYQCKQPCSCPTVCVKSAPAQTPCSPPSSCICVSEPVKVVKCPTSCQKSCTDTKAPAASCTSPCSSPCPVVCVSSCESKPACSCQTAT
CVVTTQSCDCTKPCQDTKSCAPAPEPCQTQTCTCIPVQLSQCCCGRIVLVPVSACAAPSAPPAPCCTCTCCCKPCCN

>Oh_EDCS1
MPCNCCNYCSPCCGTSTNKIICAPGQSYHVSPSCCMCGGGGCCGSCCGSCCSSCCCPPQMMCPSPMKCCCPPAMKCPPPVQLTQSCCLP
KKCC

>Oh_EDCS2
MYGGCGCGGGGGGSDGTTIICIPCGNSCGSMGCCMPCCGGMRGCCSPCCYMPCSPGGYGCGCGGIMAYRIPSSRRGSSSSCCGGY

>Oh_EDCS3
MTCCGDCCDCCGATCGNCGAVLLCVPCCGCQQCCSPCCGQSSCSSCGGQSSCSPCCGTKSSCGQSSCGGQSSCSSCGGSKSSCXGSKSS
CGQSSCSCGGSKSGCGQSSCSCGGSKSSCGQSSCSCGGSKSSCGQSSCSCGGSKSSCGQSSCGGQSSCGQSCCGGQSSCGQSCGGQSSC
GQSCCGGQSSCGQSSCGGCGQSSCSPCSCALPCIQTCCPISCPPCCKK

>Oh_EDEPK
MSTEQQQQKQTPVFPSGPSKLMAELVPEIASQKGHDGESLPSAEQGDVVPEDLKESHQKKEWTSECSAIEIPVSEDTSGAKHGSLTRKE
KEHLTSVDQHCREPPTLSQVPNKETFKSSEPPFPVREAEGHPATQPSVLGEQKQQQKKQTNQWLPK

>Oh_EDEPT
MAYQCKEPCLPPPIDVAQCSGDAQDSCTLTDMAQCVQTCDCVPLEACSAPDSLSYQTKQPCVSPSFKVALCPKAISIPCTSPCLSLRTE
MCAGSPSVPCGSVALQPGSPCPEDSQPCSTGTADACAPPSPPTCGSRCSGHCSGPQGDGGMSYVFPGSTVCLEPSGCVSVKPGVGSPEM
EGTTMFLASYQPHFM

>Oh_EDETM
MACLGLSSYNPAYSASLPSKSFLLRPPPFVAETRLTKLEILVPEHIPWDVPDVCSHPYSPLAFCYHEPPIVVGFFETSLHEV

>Oh_EDGPC
MGNMESNSGGGGGCGCSGCGCGGDYGGSDAHVICMPTGGCGCGPSCCAPCCCAPCCSPCCCAPCCSPCCCSPCCCSSGCGGQSSQCCSQ
CCKQCK

>Oh_EDGY1
MNFYTQPESIDSSYLPFYLSCYAPCYPQYGYGTGTSYCDPGNGCNYGYQPYSYGCGGLNTCSTSPCSYGYRYTDRGSCYEPWNYRMGYG
ARRNFPSRRYNCPNPW

>Oh_EDGY2
MSCYPNPSCYDPCRAKSFSSGSSGLKRRTRWSSGSCYGSYGRGVRYIPSCGYGYGGYQSPAHGGICYQPIRYDCGYGYGYGNKYCPPVC
YGGYGSGGYDYGCKYGGLYGRRQSICYDPCYGPRYQYGSRRGSFCWPC

>Oh_EDHEM
MPCTTPNKSTEEITAMHFVSIQPLDEISLGSHFHPNHHDPGIFLEDPNVMECIHFNDAHPHGEFYRRIHIVEMIELPFSPDAKESDCPL
HKHVHPGSEHSQEDPEKATSPSSSEPKSKEKLETANDGGGENIASNGDANNLSPSKDPEDAVGLMYTCKLEYQWSPMLKYCTMEELGYK
PLHLL

>Oh_EDKM
MSRLLRAFTEMIEGNSKVAPRKSTDAELLKKSEFKTLIQKELTPSKTRKHKNAKLLHESDGELMNEKELMGQWR

>Oh_EDP3
MSQQQCKQTPCVPPVSHRCTSSCSKPCEPSTCYASSGPHCPGSTGSHCPGSTGSQCPGMTGIPCSGSTGGSCQNPTCRSGNNGCFCRHP
QQQC

>Oh_EDPAM
MTHQCKLPVELPGCLKIGTKSSSQTKSSSAPSTCACCCGIPVSAPPPSPPPQTVTVCAIPAPAPAPTQSMTCCGIPVQSTSSPPPQTVS
ICAVPAPAPAQSQSITCCGIPLQTKSATPPPQTLTICVPAQAVAQSQCQSVSCCGIPVQAAKPAPAQTLTICAVPAPAPAPAPSCVPVT
LHLVQPASAPTSSCMCCSGCKT

>Oh_EDPCCC1
MTKCSKCGCSPCCCKCGCSQCCKTCCCPKCGCCPCCCCCCPKCGCCPCCCCSPCCCQQCNCAPCCCKQCVCAPCCCVCGCSPCCCQQSS
CSQSCCGQSKGCESKGCCSKCCFPKQGKS

>Oh_EDPCCC2
MTCCSKCGCSPCCCQQCCCSPCCCQQTCCQSCCEKGCGCGCGCGCKGGCGCGCXGCGCGCKGGCGCGCGCGCGCGCKGSCGCGCGCKGG
CGCGCKGGCGCGCGCKGGCGCGCGCPKQGKC

>Oh_EDPCCC3_partial
MSSGTKSPKCAKCGSSPCCCSKAASKKKCAKCGSSPCCCSKAASSLKSKCSKCGCSPCCCSKTLCSKCGCWPCCCPQSPCAKCGCXXXS
RCSRCGCSPCCCSQSRCSRCGCSPCCCSQSRCSRCGCCPCCCPQSRCCRCGCSPCCCRSQSRRCSKCGCFPCRCSQSPCCRCGCCPCCC
PQACCPRCGFPCCQQIQPYCYPCHR

>Oh_EDPCCC4_partial
MACCPICGCYPCCCVPCCSCANCCGCGPCCSSCGAPCCCSPCSSCCPCCACGGCGSPCSCPQCSSCCVCGPCCSRQSSGGGCGCGCSPC
CGCQSCGGSCGGSCGXXXCGGSCGGSCGSCGSCFPNQCFS

>Oh_EDPCS
MNYEYLFPEFLEEAEPYFFEANEDIVMPTKGGTYCPRPCPPYKCVTPRPRYCRPKYPCPCPPPCPPPCPPPCLPPCPPIRPQCPSPCYP
LYPLPQWPKPCYPSYPCRYPPQWICPKPLPCVPQRPYYPSCQLPCPPYWGRPCYPKCLPQYPCDLLEIY

>Oh_EDPKC
MASSHNQHQCKQTPTLPPEICKTAPCPPEQQCKQPPVVEVPKPKPCPPQEPPCKEPPVVVIPTPCPEPKPCPPKEPPCKEPPVVVIPTP
CPEPKPCPPKEPPCKEPPAPIPCPPEEEQCCQQKSQCKKPPVIPTPCPEPKKPQEPPCKEPPIVVIPTPTPCPEPKPCPPKEPPCKEPP
APTPCRPKEPEPCLPKEPTPDCQQKTQCKKPPVVVIPSPEPEPCPPQERPCKEPPVVVTPTPCPEPKPCPPQEPPCKQPPPCDQQQQKK
QPCSWPPQNK

>Oh_EDPQ1
MYTTENGDGEDSYSNYMRSVSCEPPSYSVRWSAEMEYCDTPPPPVRSYCPPRPIYCPPAPKGCPTKQKYCPPVQRYCPPVQKYRPPVKY
CCPPGRKSQPPAQWCSPPVQKCHPPVQSYCPPVQKYCRPVQRYCPPQQKYHPPAQPCYPPQQKYCPPAQSYRPPAQPCYPPQQKYCPPA
QSYRPPAQLCYPPQQKYCPPAQSYCPPAQPCYPPQQKYCPPAQSYCPPAEPCYPPQQKYCPPAQSYCPPAQPCYPPQQKYCPPAQPCCS
PQPKICQIKEVCKAPPHLLKK

>Oh_EDPQ2
MSYQQQQCKQPCQPPPQCQKTCPTPVCTPQVCIPKPCPPQDCIPDPCPPKVCIPRPCSPQECLPKPCPTQECLPRPCSPQECLPKPCSP
QECLPKPCPQQVCLPEPCPQDQQSCRPSQRPKRC

>Oh_EDPQ3_partial
MSYQSQQCKQPCPIPPQCKIIPPPQQCCPPPQKCCPPPQXXX

>Oh_EDPS1
MFHCCMPSCQMQPIAPLCVQPLQQPSISIPCSPPLPPMPSVSIPVSLSPPPSCIPVCRRQPSPSSSPTISIPLSSMQPPPSISLAPSQS
PSGSSPTISIPLSALIPSPPSQPCCISLCPQPSSSGPSISIPLAPSPSPSSRPPCFLPLCPGQGSPCISLVPSQPSSGSGPTISIPLSA
LQLRSSISLAPSQPSSRSSPTISIPLSALIPSPPSRPCCIPFGPSPSSSGPTISIPMNFSPRSNPSPICCFPIPMPSSPCFIC

>Oh_EDPS2
MFGYCLPSYYMGPIAPLDVQFYQQTSSMPAIGIPCRSVQSSMPPPCIPIRFSPGPSTSQSGPGSFPSCIPICPMQSPPSFSLPPYSPSP
SRQHTYCIPFDPNPSPPSSSQTQSIPLSFCPPPSSSSQTPYCIPLCPSAQSPSSGQTTSAGQPSYCIPVSTCQPSPSMSFCPAPQSSSS
RPEMSISLSPSLSSGSQSYCIPFCSYPSPSSSNQPSYSVPCCPSQTPSDANVSIPVSFSPSPSPNPPPTCRIPCSPCFLVF

>Oh_EDPS3_partial
XXXIPLSSLIPPPSKPSPPCYIPLCPSPESSNSSSGISIPLGSSSSSSNQPPCYIPICPGQSPPQPSSGSGPTISIPLSSLIPPSSSPS
QPCYIPVSPCQPSSGPTISIPMNLSPSPSSNPPPSCMIPIPMSSSPCFIY

>Oh_EDQK
MSSKDQQQKKPCGTFSTPQEPQKQCLPKNCCVVNPPETRSPCCLSPQPCTPPQKPGCLSCHPCPQNPKSYQQ

>Oh_EDQL
MCSRNNEGCSHDNTCHSGRSCCHEDRGYCGDRSNPSFTVTAIPVVQDKNPLTQTQKQCPIVYSTPQLQHTQQLKQPIPYPPQQQLKQPQ

QLKK

>Oh_EDQSG
MQQSKQPQGFPPMGQTTNQCKGSTGGQTQGPAVQQSQCQDSAPSQCQSSGVTHVHSSGVSQMQGCGVSQMQGSGVTQIQSSGLGQSQQS
DLDQCQGSGISQCQGSGGNQGKGSPMDQCGCSPVSQGKGSSGGQCKGSPVDQCGCSPVSQGKGSSGGQCKGSPVDQCGSSDVCQGKGSS
GGQVKGCPVDQCKGSKGKY

>Oh_EDSC1
MSQQQQQQGSCCHGGSSGLGGGCCRRGSSGGCCSRGTTGGGGCCGRRGSSGGCCSRGTGPVYVQQSQQTARCCCINRSGPCCGRCCGRR
CGGGCCGGGSTQLKQK

>Oh_EDSC2
MSQQQQRGGCCCCRCCGGGGGSSQVSRSSGSGCCGRSGGGSSQQAQSSGGGCCGRSGGGSSQQTQSSGGSCCGGSSQQKMK

>Oh_EDSCP
MVLSCHRPTCYSPSGPIFPVGGGSSCCVPVCHPAPCGPPTVCVQPVQSSCVPVCASPCGMSCGPPTVCVQPLQSPCVPVCASPCGISCG
PPTVCVQPLQSPCIPVCGSPCGPPAMCIQPKSSFPSCVSPSGPIFPVSSSSSIPVAPLCVQPSKPSCVSPSGPIFPVSGGHSSCMPTCA
IPVSVHPCSPCHLWF

>Oh_EDSPR1
MSYPYQQCKQPCLPPPVCGKGCATQCKVCVPACPCDVPICATPGVKVVGEACVPVCPPPCAPKCAGPAVDICVKPQGVCPCDCKCPDPC
CK

>Oh_EDSPR2
MSQQKQCCKIPPCPEACCPPKGPQKTCCSHPNKDVPQKGPCCPPSKDVKCPPQKAKQCPKHC

>Oh_EDSQ
MSYQCKQPCLPPPCLKGATVCVGPGTTVCISPTQSQGIEVCASPTGAICVTQGQSSCQCAKISHECCGPVVVTPVQTQSFSPCATIAVS
QNQGQNSTICVPQSQGCQCVTCRGSVATIQSQCGCATVCASPVCISPVAAPLAPAAVVCQDPCGNVCVASKGTAKCATKCAAPGTVVCV
DPCECVKSVKNCDDAVCAKGCQSVNQCNVKKC

>Oh_EDSRWM
MIFYTPQQYLDLDSWDINYGNDDNSPSEYFYADDPTSFVLDHKSRWDSPPYSCSSDLCGPRVRASSGSRSSCHPSCVKCHCWSPSCPDP
SCCPPWSGCGSCNPCYSPLCKPRPGRSRSAPPSMGNWGPQSSSSSCCPPWSRCGPCNPCAPCCRWGPPSMSRRGSQDSTSSCRYFPPLS
RCGPCNPCAPCCHWGPPSMSRRGSQDSTSSCRYFPPLSRCGPCNPCAPCCRWGPPSMSRQGSQDRSSCYPPRSTCGSCKPCYSPICKPR
CHSLRCPPPSMDSREPQSSSSSCCPPWSRCVPCNPCSPCCRWGPPSMSRRGSQDSSSSCRYFPPLSRCGPCNPSCPSCCWNFPSRIRWE
SQDRSSCYPPGSTCGSCNPCYSPICKPRCHSLRCPPPSMDSSCCCPPFCWSPPFMNRWGPPVCRSCPTIYYYPCYYC

>Oh_EDWM
MPEERIYSSGREAYFNLNSTWYDPAGSWLDTRRKPFRYVDNTACVTCCNPRSNVPRRGGHNYRCYCYRQCTCTPGGNPRVTCCVHNPSG
GPRDYWGRPIGDACDGCTGGHYSHAGSDCGSCCGSLGGCGTGGRTMACAQPCATSGGVCAEPGCRPAGRGVCAEPCITSSGGCSSGRGV
CAEPGCRPAGRGVCAEPCITSSGGCRTGVCAEPTCTPSGYGRRRRGVCFEPCSGTSNGC

>Oh_EDY1
MIGITILNNDPSGDTKAIYCPPTTYGPSASNPSICPPYSSLAGTAARCYIPRRYRHLFGLSPNCYLSDLSMACGYSSSLSDWRSACQPC
NYGHLFKSNQGYYDPCTFGFLSRGVQRSSDPYGLEYLYSYRRRYCDPCGYSYSPSYSRRCYDPCVYNYPSNYGRSSYPSRGYTYRCGYS
NDPCGYSCATWSCQPRYAERRRYSCQPVSCDPYGFSFESPYSSGSWRRSSDFCNDCGPY

>Oh_EDYM2
MPYYAYHCKPIGPPLPMFVKKRLPKYGSQYIPLYGSKLISSRNIPLYRRGIWVTPYSLAHVAGYQPRTIPVEFDACLNKFSKLRTTRNL
VRSDVEDYPYQIQSTVGYDGTTKGPHGPIHKGSLPCVVPERVPRMTKGPVLYSSRDLHQGLTQVPRVSRRPTFYASEDPLLCSSKEELS
QRTKRLTTQYPSKVPQGNRVRDSDLPGIDDPLQPEAKISSQPTATKGSRVITTKVPRANFLTLSHSFIKRGARSFLTKTSQAHFYRGSR
ASLVKPSRTSLVKGSQPSLPHTAIFPAPKNLSKTVKISSTGKKYSSAVNWPF

>Oh_LOR1
MASCQQSKSCCYSSGGRCVCICSGQGGSLCCCKGRGRGSVRCVSPIQQTQTSVCCGGGRRGSSGQTIIVLPGSGGGDGCCCRGRRGSSG
GRSGQIIISPGGGSGGGQSSECCIGGGICGVPGIGMNQQKCGGGIICSGGWRQQSGSAGGSCGVKVISGGSSGGRRGSGSRSRSGSPIG
RSSGGGQTIIVPCGCKGSGSRPGSPIGVCSCGDQTIIVPCPRRGSGSGSRPGSPIKGVCSGGQTIIVPCPRRGSGSGSRPGSPIKGVSA
GSQTIIVPCPRRGSGSGSRPGSPIIGGFGGGSIDGGKTIIVPCDGGSGGGGSSSVQVSGGGITVGVGAASMQTKQSIILPPCIGQTKQA
PQCPPLQKL

>Oh_LOR2
MSQQIQSSGCCSTGGGSSGHCHRRRGSGGTGCCCACCCCRGGSRHVMVVSGSQGSSCCGGIQQIPSYGCCGGSPTGVVVVPGGQSSGCC
IGGGLGGGICQQKVPLIGGDSGGGICCGGGSGGGLGGQTTIVAGGGSGSGCCGGSGVVKVIGGGSGSGSGWCGTGGGSGGQTTVITGGS
GGCCGGSGGVKVIGGGSGSGSGWCGTGSGGSGGQTTVVTGSGGGCCGGSGVKVIGGGSGSVCVDGGSEGVKVIGGGSSGGGGSFGDVKV
IGGGSGGVCGTGGGSGAKAVVVTGGSSEASACCSTGGSSGTVAVVGTGGSGGSGGKTIVVSGGGGGSDCCSGGGSGGASVVIAGGGGSS
QTKCPIVVPPCIGQTKQVCPLPPHIK

>Oh_PGLYRP3
MVILQISFLILCSLSQDLASSISPATGCFRLITPSKWEAKAANCSQPLKDVPAEYVVIHTAGNPCRTHRDCHNEVRMIQNYHMNLKGW
CDIAYSFLIGEDGYVYEGRGWRNEGSHTYGYNDLSLGIAFIGTFVERSPEDKAWKALRCFLDFSVKIGYLSPEYIMLAHSDVSDIVSPG

EFIRAEIAKWPNYKHSLYILNRGGQ

>Oh_SCFN1
MSYFLDSVCTIVGIFHKYARCQDGNLALNRREMKALIQKEFAEVLENPCDPQTIELTFKLLDVNGDSLVDFNEYLIFVFQIAKGCYRYL
QPREYLLRDESSRALHEGEAGGSKRGDHQLQDGERRGDYVHERQGLDGTFLHSTEEGSRGELVGRYLPIEEVEEEDSNFEGHEPKLRDG
DRRDHPSQEHQEREPEQQWREPKEWEDIETPEPCQWRQQESTLEEDSPRQSRELVRRNDNDRREARGLLDRDEEDLYSPIVPSRREEMR
QHRGQETEGRLFRYGSQARVDHTDYDVERSWPSHEWQEVEDGRRELEMHNERRSHAFEHRVADDPRDRIHHRSPVEEYEEHHPLSTEGE
RRSQFHEDDHRESERRRQGRSVFQSRKDVRRAFRAEEEEVRRSERRHDEDEWNRPQPREASRRSEVQDLERRERETRRPYSSELKNMDS
PSRSHESESREEVRRSQHPEDIVQRRREFSREDTRVTEVRRRRSQSLEPVARDIRYVRSQRYEEPRSNDQRRPHNYEGRSLERDVARRR
RLQLRESPKREDNQERQWYYEHETWDSERDGRRCQLRYSEPREREMRRYQVVDSRVDEREQSREAVTRPTQWDRFSPSDSPGEYDQSGQ
QIYESRHTLQREGRGQRYDRLSNGQRQIWAIDIAQRESEGQRRSNVEVRIVDQFRGVDHRESEHQRRTSIHDSDDENTDQRQTQTYEAS
SREDEDQRRTSSHDSGTRQINQRRTPTYEVGPKDDQRRRRMPSRESVDQRWRTHTQTDLAEDGQKPSNPYETDPRDGEQQRTVQSSDPN
FRDTEATQHNMGDSTQPREATSLSNQTADREFDLRRDQRPPESGESQPSRKPGQSKAEQSPVSPYEQHQPTRESQRLSGPELRGPQRRW
LPHHQTEAPEEAGVDQVQRKVGSPVSKRAISQPSERQITEGQESRIQGYHRTPVSQAEGESELSLDGEGDKAQEPAALNQQQVHREGEV
PQPVAEEPKVEEDDGSGSWAQESQPPLEDDDQQAITEKPSSSLVESKSCVICNPLYEYLLAQKKQEQP

>Oh_SCFN2
MAGLVDSICTIIAVFHKYADRKSESSSMKRRQMKRLIQKEFGDVLENPRDPQIVKLTFQLLDVNGDNRVDFNEFLFLIFEMATACYSVW
HPRECFSYNEERRRAVPDEEPRGDESNRREFLGEDRRRDDVRERRGADRTPLPSMEEGRRGELSSELREEVDRRFEGHDRELRDGDRRD
RPSRELQEREPEQRRLEPKEWEDVDIPEPRQRRQRESAVEEDSPRQSRELVRRNDNDRREARGLLDRDEEDLYSPTVISRREDERQRQG
RETEHQEAEGRLFRHGSQARVDTRNHRPEYDVERGQPSRERREGEDGRRELEMHNERRSRAFEPRVADDRRDRIRHRSPVEEYEEHRPL
SREGERRSQSYEDDHRESESRRQGRSVFQSREDVRRAIRAEEEEVRRSERRHVEDEWSRPRPREASRRPEVQDLERRERETRRPYSSEL
ENMDRPSRSHESEGREAVRRSQRPEDVERRRQELSWEETRATESRGRRPQSQEPEPRDPKYDRSQSYEEPRRDDQRRPRNYEVRSQERD
AERRRRLQPRELVEREDNQERQHYERESRDRERDGRRAMSLEAETRDEVRRRPQESASRYGERERLPEGDARMRRTGRDMASPSDAGRR
ELDPSSPQQISRRRAPELRRSEPHSFMPATKG

B

>Oh_Beta1
MSGSGVKCVTTPCVTSCPDAKVVVHPPPLVLTLPGLSLKTSPNQCLVESQTSCLTNGCEVNCDPGSKAIVTKTSGLVALSGGDTPCCTT
TCPDSQVVIQPPPVCITIPGAVLTSYPNECLISSSTNCITSGVQRPALPRSTSVSDCSLTPSRLTRSASVPSGLNSSTRCLTQGPSNKV
VIYPPPIEITIPGPVLEIAAEECTVEVYNSCTDNNALTGSDQAAITSGDESEVKTLIPRAKSCTTVSGLMDTSTCISQGPEMKIIIQPP
PIEVELPGPILEVFPEACKVETLTPCPPKPEAITGNEIKALCDSKIPSTALATTTTSTKRPLPDVRRPPRPWAEMYSRSMTPRSLALSQ
QSRLAKYRSALYSMHSQSSY

>Oh_Beta2
MDRPSPCYASCPPSTVVIRPPTFTLTIPGPAIFCPNQPLQIAQHNPCAHGGMGVEERMMLSDFINEDLPNLELELSSRYGSALATLYRS
RMAFPSESWL

>Oh_Beta3
MSECYASCPASTVTIQPPPFVLNIPGPALFCPDQAFGIEQHNPCAGFGGMGARGSGVGIEGTVGGSMGALGPGRGGWGGSWSGGSGIG
SRMGGGLGVAGVGGVGGLGGGGLGIGGPTGLGIGGLGGPRSGGLGIGGLGGPRSGGLGIGGLGGPRSGGLGIGGVVGPRGSLGLGGPR
GTTAGWSGGSRLGGXSVGMGSMTGGDLGSGEGGDMSGRRGSIGAGGSTVGWSGGSRLGGTVGGRRGSVGMGSMTGGDLGSGEGGVMSG
RRGSIGAGGSTVGWSGVGDTVGVRRGSVGTGSITEGDLGTGGVDGTIHGGLSSGGTRIITGGRGGGSGTGVSVGGRRGSIGTGGTIGG
GVGSSMSGRRGSIGTVSVTGGDYGLSGTRGSTVGGWTSGRRMSGSHRYGGSIGYGGGGYGGGYSSGGYGSSGYGDGYGHTGHGSGGYG
GGYGHTGHGSGGYRRIGYSVGGGDGYRSGGFSSRSYGGSFGGNLAVMPGPSDIYSYY

>Oh_Beta4
MSDHCSFSCPPLKVTIQPPPFIITVPGPSICCPDQPLCIEQHNPCAINHNSPPQAIRASSFYSRALPSTPYSPSIYRF

>Oh_Beta5
MWNECYAQCPASTVTIQPPPFVLTIPGPALYCPDQPLGIEQHNPCALPMPRGGSNLTSFYSRALPSTNFSSSTYRSSY

>Oh_Beta6
MASGWNCYTSCPPMMVTIQPPVYTLNIPGPSLYCADQSVCVEQCNPCAAIPSSGIINHGGYGLLPANVASDFSCSSPKSLENAYYF

>Oh_Beta7
MTSGWNYCYTSCPSMIVTIQPVPFAMNLTGPALHCPQQSLCIEQHNPCVPTPHCKDSLCSSTASNFSTSSVASQKSISRSQRSGCFPCR
WR

>Oh_Beta8
MSCGRMPCYSSCPPVTVTVQPPPFTLSIPGPSLYCPDQPLCIDQCNPCVPPPPLCPPITRRSYASSVVSSPSLPQKSLPRSQSEWSSVS
QRF

>Oh_Beta9
MASRWDLCSFSCPPTSVTIQPPPFTVTVPGPSLFCPDQPLCIDQYNPCVPQCLSYNRPRPLILASTSSQKTVSKF

>Oh_Beta10
MSYGWNNCYPNCTPVTVTLQPPPFSMNIPSSSLCCPDQYACIDPCRPCSPTVCSTMVCSPTVCGPSMCAPKACGPAACGPAHCGPVSCA

PVSCGPVSCAPVSCGPVSCGPVSCAPVSCAPVVCGPAFSRSRYNSYYNSCSLPSRKCLPRVQTICDPCTKY

>Oh_Beta11
MASKWDLCSFNCPPTTVTIQPPPFCLSIPGPSLHCPDQPLCIEQCNPCVPLPYRGSLPLPSSAASIYSQKSQQQQQQQQYQQRF

>Oh_Beta12
MHSGWDHCYPNCTPMTMMVQPPPLTLSIMSPAHYSPGHPACIKPHNPCAPVHCWDGRSHYGHHGLTQGAAPCFTSTSQKCLPKSKSGYV
PCRTY

>Oh_Beta13
MTSFNFCTCASCSSTRPCFHAKPSCLCMYFSQINMPLHQELVSTPREMLSTTQRIQGFLKQKTREYGCLSTSRPLKYLRGPTSGISYSL
QRNPPLQSYRYIKC

>Oh_Beta14
MCRENKLVMAIHPYFLDGRPNPPAVAVTIPGPILSACTEPIAITQHSPCAPSGGECAFPGYGNRSGRRYHNRIGPRSGGTQKSSSGNGG
PYLKNGKLEKIIRKSCKVFRNSENS

>Oh_Beta15
MSYCGSICHVPYASNSGSCALVSSYGSGTLTGLAANCYTGGHVNCSSQLTGSELIIQPPASVVSIPGPIISSTHDPVSVGQVTPCSYTH
PLNSYGAYGRLGYWGYGNYGNYGNYGGWRRYSRKCLTYN

>Oh_Beta16
MALCGPSCAIPSCASAPSVGFGSAGLGGLAPGSLGFSPFPLSESSGSLGTLAGIVPSCINQIPPAEVVLQPPSCVVTIPGPILSASCEP
VAVGGNTPCAVAGGFGQGLPLSLGGPSRLGARYGFVGNRGSICYTPC

>Oh_Beta17
MAFCGPSCTVPSCASAPSVGFGSAGLGGLAPGSLGLSPFPLSESSGSLGTLSGIVPSCINQIPPAEVVLQPPPCVVTIPGPILSASCEP
VAVGGNTPCALAGGFGQGLPTGLLGAGLLGSRLGRRYNFVGKRGSICYSPC

>Oh_Beta18_partial
MAYCGPACAVPSCASAPVVGFGSAGGKGLGWGLGYGGLGYGLGYGRGLGXXXGPILSASCEPVAVGGNTPCAPGGIGGIGGFGHYGGFY
GGRLGRLGRRGSICNLPC

>Oh_Beta19
MAYCGPACAVPSCASAPVVGFGSGGSKGLGWGHGYGLGYGYGLGYGYGRGLGYGYGAGALAETSGSLGTLAGVIPSCINQIPASEVTIQ
PPSSVVTIPGPILSASCEPVAVGGNTPCAPGGLRGGWGYGGWGYGGWGHGGLGFRGYGGLLGKRYPWNRRGSICLSRRGSVCL

>Oh_Beta20
MPYCGPSCAVPSCASAPVIGLGSSGCGPWGYGYRGLGLGYGAGALAETSGSLGTLAGVIPSCINQIPASEVTVQPPSVVVTIPGPILSA
SCEPVAVGGHTPCAAGGYGRYGGYYGGRLGRFGRRGSVCALPCNPC

>Oh_Beta21
MACCPPSCAVPSCASTPVVGLGSTGCGPCGYGSWGYGYGGLGLGYGYGSARFGESARNLGTLAGVVPSCISQIPASEVTIQPAPVVLTI
PGPILSASCDPVAVGGYTPCAPGGFGGYGGYYGGRLGRFGRRGSICSVGRRGSICTLPC

>Oh_Beta22_partial
MAYCGPACAVPSCASAXXXRLGRLGRRGSICALGSRGSICNLPC

>Oh_Beta23
MAYCGPACAVPSCASSPVVGFGSAGARGLGWGLGYGGLGYAGLGYGAGALAETSGSLGTLAGVVPQPINQIPASEVTIQPPSFVITVPG
PILSASCEPVAVGGNTPCAPGGIGRLGASYLGGRLGRLGRRGSIICNPCNLPC

>Oh_Beta24
MSCCAPACAVPTCIPACSSPVCYPVGGLGSLTSCGVGSYGMGSSAGGSSSAASLALAPGASVSCVNQIPPSEIMVQPTPIAVIIPGAIL
AATCEPVRVGGYTACASGSSSGGSSKLRYVPCNPCGPCK

>Oh_Beta25
MAYCGPSCAVPSCASAPAVGFGSAGARGLGWGLGYGGLGYGLGFGLGFGRGLGYGYGAGALAETSGSLGTLAGVIPQPINQIPASEVTI
QPPSFVITVPGPILSASCEPVAVGGYTPCAPGGIGRLGASYLGGRLGRLGRRGSIICNPCNLPC

>Oh_Beta26
MALCPPTYVIPSCASTPQFGLGSAGASAGLGLGGRGLGGGLQGGSISGLQGGSMGGLGGGLLGGGVSSGELGTLSGIVPQPINQIPPAE
IVIQPPSFIVTIPGPILSASSDPVAIGGNTPCAAPGSRILGRPSAPSRGLLGGRNLQEGRGNITLVPCGY

>Oh_Beta27
MAFCPPSCTIPSCASAPQFGLGSAGASAGLGLGGRGLGGGLQGGSMSGLGGGLLGGGVSSGELGTLSGITPQAINQIPPAEIVIQPPSF
IVTIPGPILSASCDPVAIGGNTPCAAPGSGILGRPSALSRGLLGGRNLQGGRGNVTLIPCGY

>Oh_Beta28_partial
MAFCPPSCIIPSCCYTSQLGLGSAGVSGGASAGGLXXX

>Oh_Beta29
MAYCGPACAVPSLASSPTVGFGSAGAGGLGYGILPYNPAASALAESSGSLGTLAGINPSCINQIPPAEVVLQPPSVLVTIPGPILSASC
EPTAVGGNTPCAISGSGIVGSDLYGNLLSGNLGLGLRRGTLLGRKSLLGSRGNICL

>Oh_Beta30
MSCCAPACAVPTYIPACSSPVCYPVGGLGSLTSCGMGSYGIGSAGGSMSAATLALAPGASVSCVNQTPPSELVIQPPPVAVVIPGAILA
STSEPVRVGGYTACASGSSSGGSSKLRYYPCIPCNPCRN

>Oh_Beta31
MSCCAPACAVPTCIPACSSPVCYPVGGLGSLTSCGMGSYGIGSAGGSMSAASLALAPGASVSCVNQTPPSELVIQPPPVAVVIPGAILA
STSEPVRVGGYTACASGSSGGSSKLRYFPCNPCGPCK

>Oh_Beta32
MSCYVPTCSVPTYVPTCAPPPSSPICYPVGGLGSLSSGGGMMSSGGSMMSSGGGGATLAASLGMAPGASVSCINQIPSSEVVIQPAPLM
LTIPGAILSASCEPVRVGGYTACATGPSGSSGRASRSMMYSCRPYICRS

>Oh_Beta33
MSCCPPACLPPCPPSCAIPSCASVPRIGLGSCGIGSGLLGYGGGGAASASSLGILPGANVGCINQIPSSEVVVQPPYFTVTIPGPILSA
SCEPVAVGGYSACAAGYGGYLGGGSGLGAICRPRRRASICKYPC

>Oh_Beta34
MSCCPPCVPCCPPSCVIPSCAARPTVGLGGCGTPSALGGFGGIGGYGGFGGFGGYGGFSSGSGPASASSLGTLAGVTPSPISQIPSSEV
VVQPPPVVLTIPGPILAASCEPVAVGGYSACAAGSFGGSYSRGLLGAGSRGICGPRRKFSICGSPC

>Oh_Beta35
MQSQCAPSCAIPSCRPVQALGSCGIGGGFGSGGFGFGGGYGMMGYGGGSGLATSASSLGLIPGVPVSCISQIPPSEVVIQPPPFSLTIP
GPVLASSCEPVAVGGYSPCSSGGYGVSGVYGSSRYGLGSSCGYLGGSGGYLGGGRSYGSKRRQSICGYPC

>Oh_Beta36
MACFNPSYTMPCATSNVGLGSCRTGPTVSGWGGYGYSGFGGSIGGGPSGMLGLTSGANFSRTSQLPPSEIVIQPPTCVLTIPEPVVTPI
TPPMVLSSSSPCSYGSLGYGYGSGYGYGSGSVGGCFRGDIMRGFQSIGPPRGRCNIYRCPC

Suppl. Fig. S2. Amino acid sequences of proteins encoded by EDC genes of the cobra (*O. hannah*). (A) Amino acid sequences of EDC proteins other than corneous beta proteins (CBP). **(B)** Amino acid sequences of CBPs, also known as beta-keratins. Amino acid residues K and Q (potential transglutamination sites), C (potential disulfide bonding sites), P, G and S are highlighted by specific colors corresponding to those in Figure 4. Stretches of X´s indicate unknown numbers of amino acid residues, that could not be predicted because of gaps in the corresponding gene sequences. Oh, *O. hannah*.

A

EDPCCC4

```
P. bivittatus gen   TATAAAAGGTCCCCCCATTCCCCAAACCCTCCATTCAGCTGGATTAACTCTCCTGGCCAC
P. regius RNA-seq   -----------------------------------------------CTCTCCTGGACAC

                                     <  intron  >
P. bivittatus gen   TTCCACTGCTTACAAAGGTAAG//TTCAGCTAGACCTTCGCTACTCATTGCAAAGATGAC
P. regius RNA-seq   TTCCACTGCTTACAAAG------------CTAGACCTTCGCAACTCATTGCAAAGATGAC


P. bivittatus gen   CTGCTGCCCCATCTGTGGCTCTTCACCCTGCGGTTGTGCCCCTTGCTACTCTTGTTCCCC
P. regius RNA-seq   CTGCTGCCCCATCTGTGGCTCTTCACCCTGCGGTTGTGC---------------------
```

B

EDSRWM

```
P. bivittatus gen   TATAAAAAGTCAGTTGAGTCCTGAGAAGTTCTAGAGATCCCTCCATACTGTCTATTGTCT
P. regius RNA-seq   ---------------------------------------------------CTATTGTCT

                             <  intron  >
P. bivittatus gen   GTGGTCCAGGTAAG//TTCAGACCCACCTTCATCCTCTCAAGATGAACTTCTACATGCCT
P. regius RNA-seq   GTGGTCCAG------------ATCCACCTTCATCCTCTCAAGATGAACATCTACATACCT


P. bivittatus gen   CAAGAATACTGGGACCTAAACAGCTGGGAGACCAACTATGAAAATAACTATGATTTCCCC
P. regius RNA-seq   CAAGAATACTGGGACCTAAACGGCTGGGAGACCGACTATGAAA-----------------
```

Suppl. Fig. S3. Alignment of RNA sequence reads versus genome sequences confirms the expression and the presence of an intron in the 5´-non-coding region of *EDPCCC4* and *EDSRWM* genes of pythons. (A) The nucleotide sequence of *EDPCCC4* in the *P. bivittatus* genome (gen) was aligned to the sequence of a *P. regius* RNA-seq read (GenBank sequence read archive, SRA, accession number ERR216300.5250820.1). (**B**) The nucleotide sequence of *EDSRWM* in the *P. bivittatus* genome (gen) was aligned to the sequence of a *P. regius* RNA-seq read (ERR216300.7157883.2). The complete sequences of the RNA-seq reads (100 nucleotides) were aligned to python gene segments corresponding to exon 1 and the start of exon 2 including the flanking sequences. TATA boxes are highlighted by green shading, splicing signals (GT and AG) at the ends of the intron are highlighted by blue shading, and start codons are highlighted by yellow shading. Hyphens were introduced to maximize the alignment. Red letters indicate identical nucleotides in both sequences.

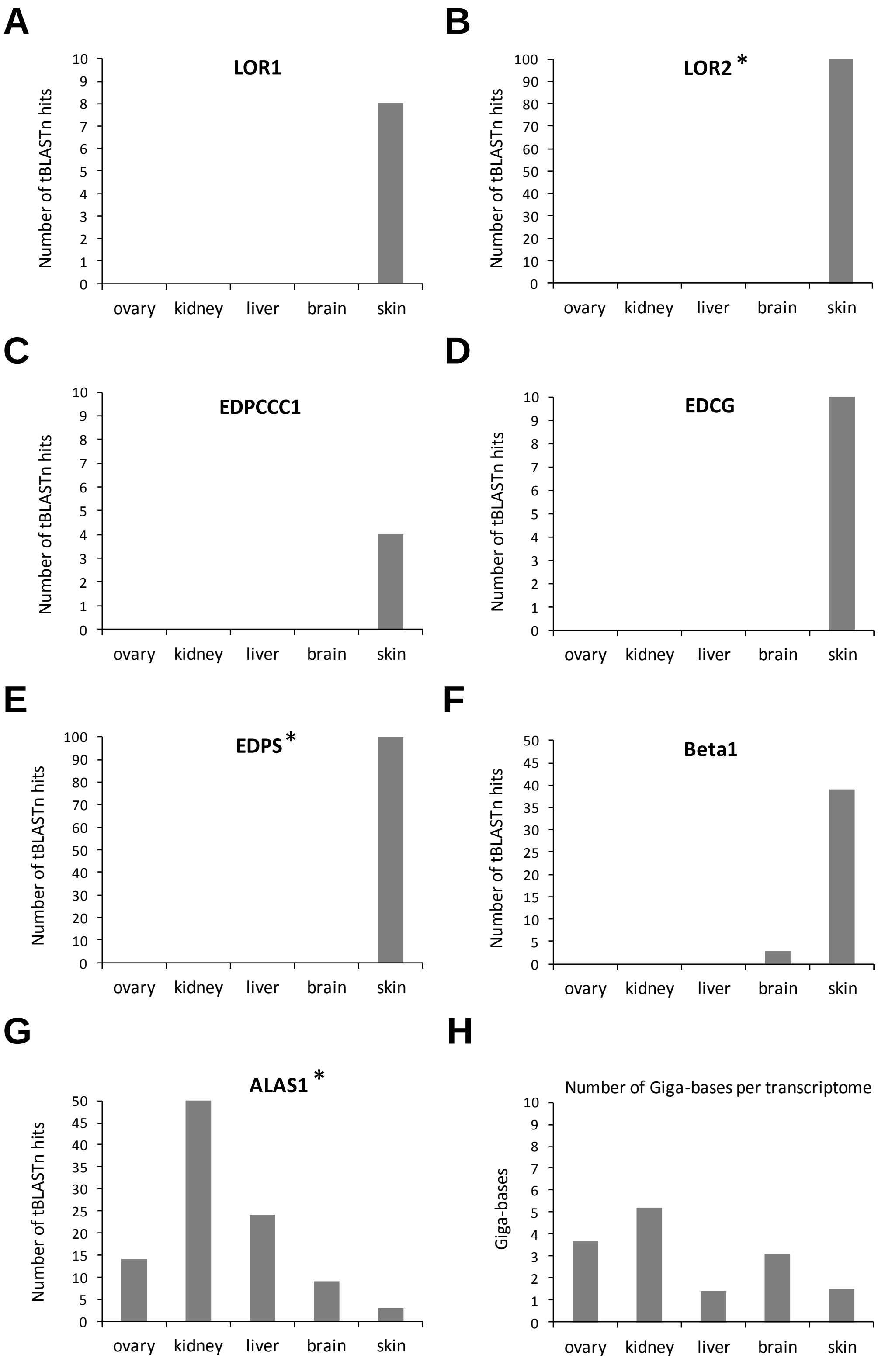

Suppl. Fig. S4. Identification of EDC gene transcripts in tissues of the painted saw-scaled viper (*Echis coloratus*). (A-F) Sequence segments of proteins (Suppl. Fig. S2) encoded by genes within the EDC were used as queries in tBLASTn searches in the tissue transcriptomes of *E. coloratus* (Hargreaves et al., Genome Biol Evol 2014;6:2088-2095). Accession numbers: SRX566773 (brain), SRX566774 (kidney), SRX566777 (liver), SRX566778 (ovary), ERX190966 (skin). **(G)** RNA-seq reads of the house-keeping gene ALAS1 (located outside the EDC) were investigated as a control. Default settings of tBLASTn at the GenBank server were used. The results help to determine the tissue in which each gene is predominantly expressed. These data do not allow quantitative comparisons of expression levels of different genes. *, only the top 100 hits of the tBLASTn search were counted for LOR2, EDPS, and ALAS1. **(H)** Number of Giga-bases per transcriptome.

CBPs with 4 beta-sheets

This study, Fig. 3

```
Python Beta1 sheet 1    PCITS-CPDAKVVVHPPPLILTLPGLSLRTSPNQCLLETQTSC
 Cobra Beta1 sheet 1    PCVTS-CPDAKVVVHPPPLVLTLPGLSLKTSPNQCLVESQTSC
Lizard Beta1 sheet 1    PCVTK-CPDAKMVIHPPPLAITLPGLRLTTNPGKCLVETQTSC
 Gecko Beta1 sheet 1    PCVTS-IPDAKVIIHPPSLKLTLPGLILATSPGKTLVETETAC

Python Beta1 sheet 2    PCCTTTCPDSQVVIQPPPVCITIPGAVLTSYPNECLIKTSTPC
 Cobra Beta1 sheet 2    PCCTTTCPDSQVVIQPPPVCITIPGAVLTSYPNECLISSSTNC
Lizard Beta1 sheet 2    PCSTTACSDSQLVIYPPPVCIMIPGAMLSSHPNECLIESSIPC
 Gecko Beta1 sheet 2    PCCISTTPDSQMVIKPPPVCITIPGAVLTSFPNECLIATSQPC

Python Beta1 sheet 3    RCVTR-GPTTKVVIHPPPIEVTIPGPVLEIAAQECAVEVYNPC
 Cobra Beta1 sheet 3    RCLTQ-GPSNKVVIYPPPIEITIPGPVLEIAAEECTVEVYNSC
Lizard Beta1 sheet 3    PCVTQ-GPGSKVIIHPPEIEICLPGPIVEIMAAECAVEVYNPC
 Gecko Beta1 sheet 3    ACVAQ-GPSSKVVIYPPPIELIIPGPLLEIEAEECAVEVHNPC

Python Beta1 sheet 4    SCISQ-GPEMKIIIQPPPIEVELPGPILEVFPEACKIETLNPS
 Cobra Beta1 sheet 4    TCISQ-GPEMKIIIQPPPIEVELPGPILEVFPEACKVETLTPC
Lizard Beta1 sheet 4    SCVSE-GPELRIVVKPPPIEVDMPGPILQVFPETCKVEVLNSG
 Gecko Beta1 sheet 4    PCVSQ-GPEMKITIQPPPIEVDLPGPILRVFPEECKVETFSPC
```

CBPs with 1 beta-sheet

Fraser & Parry 2014

```
Beta-sheet consensus    PCVRQ-CPDSEVVIQPPPVVVTLPGPILSSFPQNTAVGSST
```

CBPs with 1 beta-sheet

Calvaresi et al. 2016

```
Chick feather CBP (Fe)  PCVRQ-CQDSRVVIQPSPVVVTLPGPILSSFPQNTAAGSST
Turtle shell CBP (Tu)   PCVRRQCQDSEVVIRPSPVVVTLPGPIMSNFPQHSGVGAVG
Snake scale CBP (Sn)    SCINQ-IPASEVTIQPPAVVVTIPGPILSASCDPVAVGGYN
Lizard CBP (HC10)       SCITQ-LPASEMVIQPPAVVVMTIGPIMSASCEPIAVGGTT
Gecko scale CBP (Ge)    SCINQ-IPPSEVTIQPPPCTVVVPGPVLAASCEPLRVGGYT
```

Suppl. Fig. S5. Comparison of amino acid sequences in the beta-sheet regions of corneous beta-proteins (CBPs)/beta-keratins with 4 beta-sheets and CBPs with 1 beta-sheet. Proteins encoded by the *Beta1* gene (first gene of the CBP gene cluster within the EDC) of squamates are predicted to form 4 beta-sheets. The sequences shown here correspond to the core sequences indicated in Fig. 3. All other CBPs are predicted to form only 1 beta-sheet. For comparison, the consensus sequence of the beta-sheet region of CBPs, as reported by Fraser and Parry (Fraser, R. D. & Parry, D. A. Amino acid sequence homologies in the hard keratins of birds and reptiles, and their implications for molecular structure and physical properties. *J. Struct. Biol.* 188, 213-224. 2014) and core sequences of selected CBPs, as reported by Calvaresi *et al.* (Calvaresi, M., Eckhart, L. & Alibardi, L. The molecular organization of the beta-sheet region in Corneous beta-proteins (beta-keratins) of sauropsids explains its stability and polymerization into filaments. *J. Struct. Biol.* 194, 282-291. 2016) are shown. Each sheet is composed of anti-parallel beta-strands that are separated by tight turns. Predictions of beta-strand-forming residues are indicated by yellow shading. Proline residues (green fonts) are critical for the formation of tight turns. There are uncertainties in the number of residues in individual beta-strands, and the number of beta-strands per beta-sheet differs in the various models (5 beta-strands per sheet in the model of Fraser & Parry and in Fig. 3; 4 beta-strands per sheet in the model of Calvaresi *et al.*). Cysteine residues (red fonts) are involved in the formation of disulfide bonds which have not been integrated in the models discussed here.

A

>Ac_EDCATM
MFLSQLSCLLPPIFPTKPTGACSTQCMNSGTENRTSQYLDPCTAEHKSPSDSNFVSIPDPCTGVSAPKCVDLCGPEPKKSSQKCEDPSA
IAKDRLESSWGERWGIIINNNNDNGPQFPSSSAKQCTSTIRCPTKCTSMLPCTTKAYDSCATCVPRIILACARRCGIPCSNTCAA

>Ac_EDCS1
MGNCNGYGGGQSQPSMMCLPLGGCGSCCQPSCGQSCGSSGCLYLVSMGRSNSGGCCGGCCGGGGY

>Ac_EDCS2
MGQCGCCGGSGGGSMLVFTISCNPKCPPRSCPPQCCPPPYCPPPYCPPRSCPPPYYPQRYCPPSYRSSSCCSSSCCSSPCIYFIRLGGS
SNSGCCGGCGCCN

>Ac_EDCS3
MCSCGQSCKQSCCPPPPCCKSSCCAPPPCCQSSCCAPPPCCKSSCCAPPPCCKSSCCAPPPCCKSSCCAPPPCCKSSCCAPPPCCKSSC
CAPPPCCKQSCCSPPPCCAPPPCCKQSCCSPPPCCSPPPCCAPAPCCKQSCCSPPPCCSPPPCCAPAPCCKQSCCSPPPCCQSSCCAPP
PCCKSSCCAPPPCCKSSLLCTTSLLQILLLCTTSLAANPPAVHHLPAANPPAVHHPPVARARNAKGPTQVQGSLEIFHLSLNHVHHVCF
LAHLNCPNLLTWLLQPYL

>Ac_EDETM1
MVFPGEFSGCNTSYSTSLPPSTVLIQPPSYVAETSLTDLEIILPEPSPFEHPEYGFHNYHVPPLYCHLPPEQPLFLASFLTVLHQV

>Ac_EDETM2
MVFPGEFSGCNTSYSTSLPPSTVLIQPPSYVAETSLTDLEIILPEPSPFEHPEYGFHNYHVPPLYCHLPPEQPLFLASFFTVLHQV

>Ac_EDGPC1
MGNSGSYGCQCGSPYDCCSPACDQCCQSCCGQSCCAPACPSCCSPCQSTCPCCTPPCQQCCK

>Ac_EDGPC2
MGNSGSGGGCDCCGGGGGGESMMIVSCPGNNNCCPSPCCCAPRCCVPCCNQCCTQCCAPCCCQGCCK

>Ac_EDGY1
MGFYGGLDCYPPYFQPCYPPYYQPYYPQYGYGYGGYGYGYGNQGPCNYPQYGCGYGGYGYGSRYGSPGLQPCSYGYRYTNRGGCYDPRA
FRCGYGSRRSFPSRRYGYGGSYGGGCYGGYCGFF

>Ac_EDGY2
MTHIKGQSYCDPSGDKVYSGSSSGAYRQRNRRGSTGSYSGEYSQGRGCGDYGCYGGQGGQRNSDQGGYGYGGQRGRCYSGQEGYSYDGQ
RGRHYSGQESYGCDGQRGRRLSGQGGYSYDGQRARRFSGQGGYGYYGQGGNCYNNQGVYGYGGRQGGRHYSIQGGCGYGRQGGYGFGNQ
GDFGYGGLGGYGFGNQGGCGGLVGYGLGNQGGCGGLGGYGLGNQRGFGYGGLGGYGCLGGYGYGDQGRGTCLQPIGYGCRSEGYRYGNF
GCHSLGRGLLGRGCYGNTSSYGSGGYGYGSDGCGVRALLGYRHNSYQPGYSCGYPGN

>Ac_EDHEM
MLFLSLERSPPLLGTMDAQDCPSDTKPFKPTNFLSRTPRILIIRKQTSEEVTGNGDALVPDGNPNLVEVEREPPEKIQGNLPSVFCGND
SNGMGQEDPTPKEGSGDNDGGHGSVNLAATEGKPMEEMANHDHNSLPCANENCGIMTWRDCDIQQCKTDWRMGENIEAVLNWSGNRAST
GYDKAASYGYEGNNGSPESNDITITRPIVQQWSMDYRWSPLLKYCKPVQGHHECVSED

>Ac_EDP3
MSHQHQQQCVQPPSFPPVACVQDPKCVEPCPPPACQVVTDCQWPGSTGKPCQGSTGSSEQCVSGNHDGSASCVLQQEPKQC

>Ac_EDPAML
MARRKRSIVPPPCCTESEAKDIPSGDTPCESPCPSFTCNQKSPGAECTSLVEDTCCPEPTTADSLACQRYYCNAICGKKQVLALAGATV
QTISCSTLRPKPKKRGSYSTLRGSPTCGSPLQTSPCAHTCRIPLRLSPCNNRPRLPLQFTLCNTPSDPCSTLCSNTCNGQCNTPSSCPQ
SCTSCDEPLVMTAYLPITIRLCQATCSSNVPCVCASPCQSPTCSQCCQK

>Ac_EDPCCC1
MAYNYCHTSSPCCELFPCQKSGSPCYSHPCHKQCCDLCPKQCCDPYPKQCCSHPCCDPCCCPPHCKPQCCHPYYCPNPYNP

>Ac_EDPCCC2
MAKATSCSQCNCSPCCCEQCCCIPCESCACCPHCGVCPCSCRNRHASSDSYSCLCHECGYPN

>Ac_EDPCCC3_partial
XXXPCCCSKCSKCGCSPCCCPKCSKCGCSPCCCPKCSKCGCSPCCCSKSPCYQCKCSPCYSSKTPCSKCGCSPCCCPKCSKCGCSPCCC
PKSPCSTCGCSPCSCSKCSKCGCSPCCCPKSPCSTCGCSPCSCPKCSKCGCSPCCCPKSPCSNCESSPCCCYTHVKYSIYVPCCCPRCV
CARSALNRSCCLPCYRGQCYYNYSPCCCYGHSCGCGIY

>Ac_EDPCCC4
MTYGCGSCCNSCGYRNFCGPCCAPCCNSGCCHSGCCHSGCCSCCSPCCCSPCCCSPCCCSPCCCSPCCCSPCCSPCCSPCCNPCCGGWG
CGSCCHPCCGY

>Ac_EDQK

MSSKDQQKKQQSQKPPEEQKASKNECPEKSPKTQKKQEKEEPTKKKQN

>Ac_EDQM
MCSRQDKDKAYKQEKEEKPSGCGRQNSGNESRGCGSGRQNSGSSCGKPQKPKPSQDQQQQQQQQQVCKVPDQKQK

>Ac_EDQSG
MSYQREQQYKPMYPPACCPKPYSPPEYSRPCPPSSSSGSTGIQCQGSSGTHCRWSTRIQSSSGTQCQESSGNRRQWFTGNPCQGSTWNS
CQEPSGTQYQGPTGSLWQCSSGTDGGTHSQYYHHYRQGSQDPKQC

>Ac_EDSC2
MSQQQQRGSSCCCSGGSSGGGGGGGGGGCCGRSGGSQQSQSSGGCCRSRRSSGGCCSRGGSSQQSQGSSGGCCGRSSGGSSGGCCGGSS
GGGSSQKKMPIQQMK

>Ac_EDSRWM
MTYYGCLGYCDPCNYSLPEKCSASNGYQRLYIQILPSEYYACSPCWDNYGHQISCVFPWYRMRFSYCSPCYSYNCCSPSYYCDPFCHTL
KSSDYIPRYKSTSRYCLPHGSYNYSPSYGPKTRCYSPYYSSKTKGCSHSSSNHQFTHRYGSPSSYCSPCYRTETIRGTPCYSSNECSSC
YSSPLSSCSSCYDAKTKAWSPCYSSNESSSCYGSPSSSCSSCYAFKTKGCTPCYNNHQHSPCHRCCSPYYTSRSRCCSPGYTYRRCSPC
CSSKGYSPHYNSGSKCRTPSYRYSQCLSCYRSRCHSPYYHFGSRCGAPGYRNSHYYPCRGADFGCHSPSHSSTCSSSHSTSCTYSMKSN
SYSSGCRRCSCPW

>Ac_EDY1
MAGFILFGPYPPFDLQKIYGSQGLYHPWYEPMYYEPWSYTDPSDVFANCYVPGYYNGLPNYNTSFLQPNSYGAFPGREPNGPDPLSNNG
GSGYKPRSYSHLSGLSRNGYQPGDSGNRGSNSKPHHFGSPTSDGDRYLNLYDSGCYRPRAYLSNSSRRCYDPCGYNFNSGLSNPCGYPY
LSGCGGSCYELPYSYGYPFSYGGSCYNPCGYNYPTSYGGRYCRPRGYTYISGYGGSCYDPYSAMCPYPYYTGRNRYFS

>Aca_EDYM1
MTNCNYQCWPQNFPPQGPQFGPGPQCGPGLQCGPGPQCGPGPQCGPGPQCGPGPQYGMMKPSASFMNMNQPPFMNMNQPANFPEPYKMN
FCNIQGGMPCAPCGPTPCDSKYAESSDAKSTEKHTSSCDTSGHESCTMKNSSQPTDRCSSQRPPCPSMPGISLCSPSCGPSMGRPHGFP
SSSQCFPPRNMYQYTTSKTFKSCYAK

>Ac_EDYM2
MPYYRYYSKPLGPPLSPFVKKRLPKYGSQYIPFYGPRYFSSRTLPLYEKGLFSSKTLPLYQRGLYIPPYHQSGGAGYQPRGLNPRPESH
GTKLPQRNLARNPTQPHVNIYQRHLLQSPYSGMTKDRHGTITKGSVPGAPRAPLPRMTKGPVLYSPKDLEPHLPKAPLPRVTKGPVLYS
PRDLEPHLPKSPPPHIPKSPTKHSAKRIPPVVPTDSQLNVTKAQQHHTSKSMLSRATKGSRTIASKVPRFNLLNFSRSLRKKASRRNRA
KTSQPSLVKGSQPSLVRDSRGSLAKSSQPNLVHTATCPDLKKLSSNVKVSKTGKKYCSAAKWPF

Suppl. Fig. S6. Update of amino acid sequences of proteins encoded by EDC genes of *Anolis carolinensis* (Ac). (**A**) Newly identified amino acid sequences of EDC proteins in this study. Other EDC genes have already been reported by Strasser et al. (2014) Mol Biol Evol 31:3194-3205. Amino acid residues K and Q (potential transglutamination sites), C (potential disulfide bonding sites), P, G and S are highlighted by specific colors corresponding to those in Figure 4. Stretches of X´s indicate unknown numbers of amino acid residues that could not be predicted because of gaps in the corresponding gene sequences.

A

```
Pb EDSPR1    MACPY--QQCKQPCLPPP
Pb EDPSQ     MDCCS--QQCKQPCLPPP
Pb EDSPR2    MS------QCKQACKAPP
Pb EDCP      MSF-----QCKQACPCPS
Pb EDEPK     MSTE--QQQRKQTSVLPP
Pb EDEPT     MAY-----QYKQPCPPPP
Pb EDQSG     M------QQCKQPQGFPP
Pb EDP3      MS---QQQQCKQIPCTPP
Pb EDPQ2     MSYH--QQQCKQPCQPPP
Pb EDPQ3     MSYLN--QQCKQPCPIPP
Pb EDSQ      MSY-----QCKQPCLPPP
Pb EDPAM     MTH-----QCKLPPELPP
Ac EDCP      MSY-----QCKQRCLPPP
Ac EDPQ2     MSY--QQQQCKQPCQPPP
Ac EDPQ3     MSSDSF--QCTQPCKAPP
Ac EDSPR2    MS-----QQCKQGCKAPP
Ac EDSQ      MSY-----QVKQASLPPP
Ac EDEPT     MSY-----QARQPCTAPP
Ac EDP3      MSH-QHQQQCVQPPSFPP
Ac EDQSG     MSY-QREQQYK-P-MYPP
Ac EDSPR1    MACPH--QQCKQPCLPPP
Ac EDPSQ     MYC--TDQQCKQACLPPP
Hs Lor       MSY-----QKKQPTPQPP
Hs PRR9      MSFS--EQQCKQPCVPPP
Hs SPRR1A    MNS---QQQ-KQPCTPPP
Hs SPRR1B    MSS---QQQ-KQPCTPPP
Hs SPRR2A    MSY--QQQQCKQPCQPPP
Hs SPRR2B    MSY--QQQQCKQPCQPPP
Hs SPRR2D    MSY--QQQQCKQPCQPPP
Hs SPRR2E    MSY--QQQQCKQPCQPPP
Hs SPRR2F    MSY--QQQQCKQPCQPPP
Hs SPRR2G    MSY--QQQQCKQPCQPPP
Hs SPRR4     MSS--QQQQRQQQQCPPQ
Gg EDQCM     MSYY---EQCKQPCLPPP
Gg EDPE      M-------QCKQEVTLPP
Gg EDYM1     MSYWY---QYKQQCFIPS
Gg EDP3      MSSH---QQ-KQQQQIPA
```

B

```
Pb EDPKC     QQKKQPCS----WPP---QNK*
Pb EDQL      QQKKQGCQ----LPP----QK*
Pb Lor1      Q-TKQPISIPPCIGP-T---K*
Ac EDCQ2     QQVKQP----TQWPP--QNAK*
Ac EDQL      QQVKQP----TQWPS--QNQK*
Ac EDEPK     QQRKQP----STWPL-----K*
Ac EDPKC     HQKKQPC----YWPH----HK*
Ac Lor1      Q-TKQ----MNTWPSG---QK*
Gg Lor1      QQT-QPIS----WPPQT-KHK*
Gg EDGH      QQIKQ----SSQWPPS--QKK*
Gg EDPE      QQVKQP----SPWPLT---QK*
Gg EDQL      QQIKQP----VQWPT--QQQK*
Hs Ivl       QQ-KQ----EVQWPP---KHK*
Hs Lor       QQ-KQ----APTWPS-----K*
```

C

```
Pb EDPSQ     GQK--YCSASNNWPW*
Pb EDYM2     TGKK-YCSTT-KWPF*
Ac EDYM2     TGKK-YCSAA-KWPF*
Gg EDQrep    HAKK-YCSAS-KWPW*
Gg EDYM2     HSKKSRC-AS-KWLW*
```

Suppl. Fig. S7. Conserved amino acid sequence motifs of snake SEDC proteins. Amino acid sequence alignments of motifs present at the amino-terminus **(A)** and carboxy-terminus **(B, C)** of some but not all SEDC proteins of the lizard (*Anolis carolinensis*, Ac), chicken (*Gallus gallus*, Gg) and human (*Homo sapiens*, Hs). The amino acid sequence motifs shown in A and B were discussed in a previous paper (30). The present study shows that these motifs are also conserved in several SEDC proteins of snakes, represented here by the python (*Python bivittatus*, Pb). Panel **C** shows a newly identified sequence motif at the carboxy-terminus of proteins that are encoded by gene neighbors of the beta-protein gene cluster. *, end of the protein.

```
         1                                                                                                         111
Pb_EDPS1 MFHCCVPSCSMGPTAPLCFQPCQQSRSMPTISIPCRPVHPSMPPTSIPINLCPVPSMPP-------PSSSPSICIPLCPSPSPSSQRQPSIPLCLSPQPSGSSSGSNSRIS
Oh_EDPS1 MFHCCMPSCQMQPIAPLCVQPLQQ----PSISIPCSPPLPPMPSVSIPVSLSPPPSCIPVCRRQPSPSSSPTISIPLSSMQPPPSISLAPSQ-----------SPSGSSPT
Ts_EDPS1 MFHCCMPSSQMGPVSPLCLQPLQH----PSISIP------PMSPISIPLSLSTSPSGIPL-----SPSSSPSISIPLSALQPSPSISLAPSQ-----------PSSGSSPT
Pb_EDPS2 -------------------------------------------------------XXXXXXXXXXSSSGQSQPSIPLCFSPQPSGSGSG----------------------
Oh_EDPS2 MFGYCLPSYYMGPIAPLDVQFYQQTSSMPAIGIPCRSVQSSMPPPCIPIRFSPGPSTSQS-----GPGSFPS-CIPICPMQSPPSFSLPPYS-------------------
Ts_EDPS2 MFGYCPPSYCMGPIAPLDVQFYQRSSSMPTITIP---VQSSMPPPCTPVRFSPGPSMSPS-----GPSSFPS-CVPICPMQSPPSFSYPASP-------------------
Pb_EDPS3 MSSCCV------------------------------------------------------------PSCRVGPIAPLRVQPRDQSSSLP----------------------
Oh_EDPS3 -------------------------------------------------------XXXXXXXXXXIPLSSLIPP-PSKPSPPCYIPLCP----------------------

         112                                                                                                       222
Pb_EDPS1 ISLSPSPSSSSQSQLSIPLCLCPQPSSSSSGSSSSPSISISFSPNQSSSSQPLYCIPLSPSQPPPSISLSPSQSQSSSSHSICIPFCPSPLPSSSSQPSFCIPLRPSPQTS
Oh_EDPS1 ISIPLSALIPSP--PSQPCCI----SLCPQPSSSGPSISIPLAPSPSPSSRPPCFLPLCPGQGSPCISLVPSQPSSGSGPTIS-------IPLSAL--QLRSSISLAPS-Q
Ts_EDPS1 ISIPLSALIPSSSNPSPPCCI----SCCPQPSSSGPRISISLAPSPSPSSQPPCFLPLCPGEGSPCVSLVPSQPSSGSGPTIS-------IPLSALIPSPSSQPCCIPL-G
Pb_EDPS2 ---------------------------SGSGSGSSPSISISLGPSHSSSGQSQPSIPLCFSPQPSGSG-------------------------------------------
Oh_EDPS2 ------PSPSRQHTYCIPFD----PNPSPPSSSQTQSIPLSFCPPPSSSSQTPYCIPLCPSAQSPSSGQTTSAGQPS--------YC---IPVSTCQPSPSMSFCPAPQ--
Ts_EDPS2 ------PPVSRQHTYCIPFC----PSSSPSSYSQSQSVPLSFCPPPSSSSQIPYCIPVCPSAQSPSSGQRPSGGQPS--------YC---IPVSTGQPSPSLSFCPSPQ--
Pb_EDPS3 ---------------------------SCI---SCRPVQPSMPPTSIPISLSPVPSMSQPGPSPLSSC-------------------------------------------
Oh_EDPS3 ---------------------------SPESSNSSSGISIPLGSSSSSSNQPPCYIPICPGQSPPQPS-------------------------------------------

         223                                                                                                     331
Pb_EDPS1 SSSSSSSPRISISLSPSPSPSGQSQSCIPLCFSPQPTGSGSGSGSSPRISISLSPSPFSSSQSQPSYCIPLCPSQPPP----SMSPRPAPPPTCCIPIIIPSNPCFCGC
Oh_EDPS1 PSSRSS-PTISIPLSALIPSPPSRPCCIPFGPSP--------SSSGPTISIPMN--FSPRSNPSPICCFPI-------------------------PM--PSSPCFI-C
Ts_EDPS1 PSPPSSGPTISIPLSALIPSPPSQPCCIPLGSSP--------PSSGPTISIPLSSLIPPPPQPSPSCYMPLCPSQPSSGPIISIPMSSHPPPACCFPM--PSSPCFI-C
Pb_EDPS2 ---SGSGSGSSPSISISLGPSSSSS-----------------SQSQPFYCIPLCPT---QPSP----------SM---------SPSSSSQPTCCIPISIPSNPCFCVC
Oh_EDPS2 --SSSSRPEMSISLSPSLS-SGSQSYCIPFCSYPSP-----SSSNQPSYSVPCCPS---QTPSDANVSIPV--SFSP-------SPSPNPPPTCRIPC----SPCFLVF
Ts_EDPS2 --SSCSGPVMSIPLSPSPS-SGTQCYCIPFCSAPSP-----GSSNQPSYCIPFCPS---QPPSGASVSIPM--GFPP-------SPSPNPPPACCIPC----RPCFLDF
Pb_EDPS3 ---IPLCPMQSPP-SMSLSPSPPPP-----------------TSSQPSYCIPLCPS---QPPP----------SM---------SPTPSPPPSCCIPITIPSTPCFIVF
Oh_EDPS3 ---SGSGPTISIPLSSLIPPSSSP--------------------SQPCY-IPVSPC---QPSSGPTISIPMNLSP---------SPSSNPPPSCMIPIPMSSSPCF-IY
```

Suppl. Fig. S8. Alignment of EDPS amino acid sequences of snakes. Amino acid residues K and Q (potential transglutamination sites), C (potential disulfide bonding sites), P, G and S are highlighted by specific colors corresponding to those in Figure 4. Stretches of X´s indicate unknown numbers of amino acid residues, that could not be predicted because of gaps in the corresponding gene sequences. Oh, *Ophiophagus hannah* (king cobra); Pb, *Python bivittatus* (Burmese python); Ts, *Thamnophis sirtalis* (common garter snake).

A

SCFN1

```
O. hannah genome seq. TATAAAAGGGGTTTGGCTTCCTGGCGTTTCCATACTTTTGTCTCTGAGGCTGCTCACCTG
E. coloratus RNA-seq1 ------------------------------CACACTTTTGTCTCTGAGGCTGCTCACCTA
E. coloratus RNA-seq2 ------------------------------------------------------------

                                     <   intron   >
O. hannah genome seq. ATTTTGCTGAGCTGGGTGAG//TGCAGAAAGGGTCTTCAGGATGAGCTACTTTCTGGACA
E. coloratus RNA-seq1 ATTTTGCTGAGCTGG------------AAAAAGACTTCAGGATGAGCTACTTTCTGGAAA
E. coloratus RNA seq2 ------------------------------------------------------------

O. hannah genome seq. GTGTCTGTACCATTGTTGGAATCTTTCACAAGTATGCCCGATGCCAAGATGGCAACCTCG
E. coloratus RNA-seq1 GTGTCTGCACCATTGTCGGAAT--------------------------------------
E. coloratus RNA-seq2 ------------------------TTCACAAGTATGCCCGATGTCAGGATGGCAACCTCG

                                                                                 <
O. hannah genome seq. CTCTCAACCGGAGAGAAATGAAGGCGCTTATCCAGAAAGAGTTTGCTGAAGTCTTGGAGG
E. coloratus RNA-seq1 ------------------------------------------------------------
E. coloratus RNA-seq2 CTCTGAACCGGAGAGAAATGAAGACGCTTATCCAGAAAGAGTTTGCTGAAGTCTTGGAG-

                        intron  >
O. hannah genome seq. TGAG//TCCAGAATCCTTGCGACCCTCAGACAATTGAACTCACTTTCAAGCTGCTA
E. coloratus RNA-seq1 ------------------------------------------------------------
E. coloratus RNA-seq2 -----------AATCCTT------------------------------------------
```

B

SCFN2

```
O. hannah genome seq. TATAAAAAGGAATCGGATACCTGCTATTCTCCAACAGTTCCCTGAAGGCTTCCAACC
E. coloratus RNA-seq3 -----------------------------------------CTGAAGGCTCCCTGCT
E. coloratus RNA-seq4 ---------------------------------------------------------

                                         <   intron   >
O. hannah genome seq. GTTGAATGTACTGAGCTGGGTGAG//TAGAGACAAAGTCTCCAACATGGCTGGTCTC
E. coloratus RNA-seq3 GTTGAGCATACCGAGCTGG------------GCAAAGTCTCCAACATGGCTGGTCTC
E. coloratus RNA-seq4 ---------------------------------------------------------

O. hannah genome seq. GTGGACAGTATCTGCACCATCATTGCTGTCTTTCACAAGTATGCTGACAGGAAGAGT
E. coloratus RNA-seq3 GTGGACAGTATCTGCAACATCATCGTGGTCTTTCAGAAG------------------
E. coloratus RNA-seq4 --------------------------------------------------------G

O. hannah genome seq. GAGAGTTCCTCCATGAAGCGAAGGCAGATGAAAAGACTCATCCAAAAAGAGTTTGGT
E. coloratus RNA-seq3 ---------------------------------------------------------
E. coloratus RNA-seq4 GAGTGTTCCTCCATGAAGCGGAGGCAGATGAAAAGACTCATCCAGAAGGAATTTGGT

                                  <   intron   >
O. hannah genome seq. GACGTTCTAGAGGTAAG//CCCAGAACCCTCGTGATCCTCAGATTGTCAAGCTGACC
E. coloratus RNA-seq3 ---------------------------------------------------------
E. coloratus RNA-seq4 GAAATTCTAGAG------------AACCCTCGTGACCCTCAAATTGTCAAGCTG---
```

Suppl. Fig. S9. Alignment of RNA sequence reads versus genome sequences confirms the expression and the presence of 2 introns in the *SCFN1* and *SCFN2* genes of snakes. (A) The nucleotide sequence of the *scaffoldin 1* (*SCFN1*) gene of the king cobra (*O. hannah*) was aligned to RNA sequence (RNA-seq) reads of *E. coloratus*: RNA-seq1 (GenBank sequence read archive, SRA, accession number ERR216301.7454688.2) and RNA-seq4 (ERR216301.7454688.1). **(B)** The nucleotide sequence of the *scaffoldin 2* (*SCFN2*) gene of *O. hannah* was aligned to RNA-seq reads of *E. coloratus*: RNA-seq3 (ERR216319.8005522.2) and RNA-seq4 (ERR216319.8005522.1). Green shading highlights TATA boxes, blue shading splicing signals (GT and AG) at the ends of introns, and yellow shading start codons. Hyphens were introduced to maximize the alignment. Red letters indicate identical nucleotides in 2 sequences.

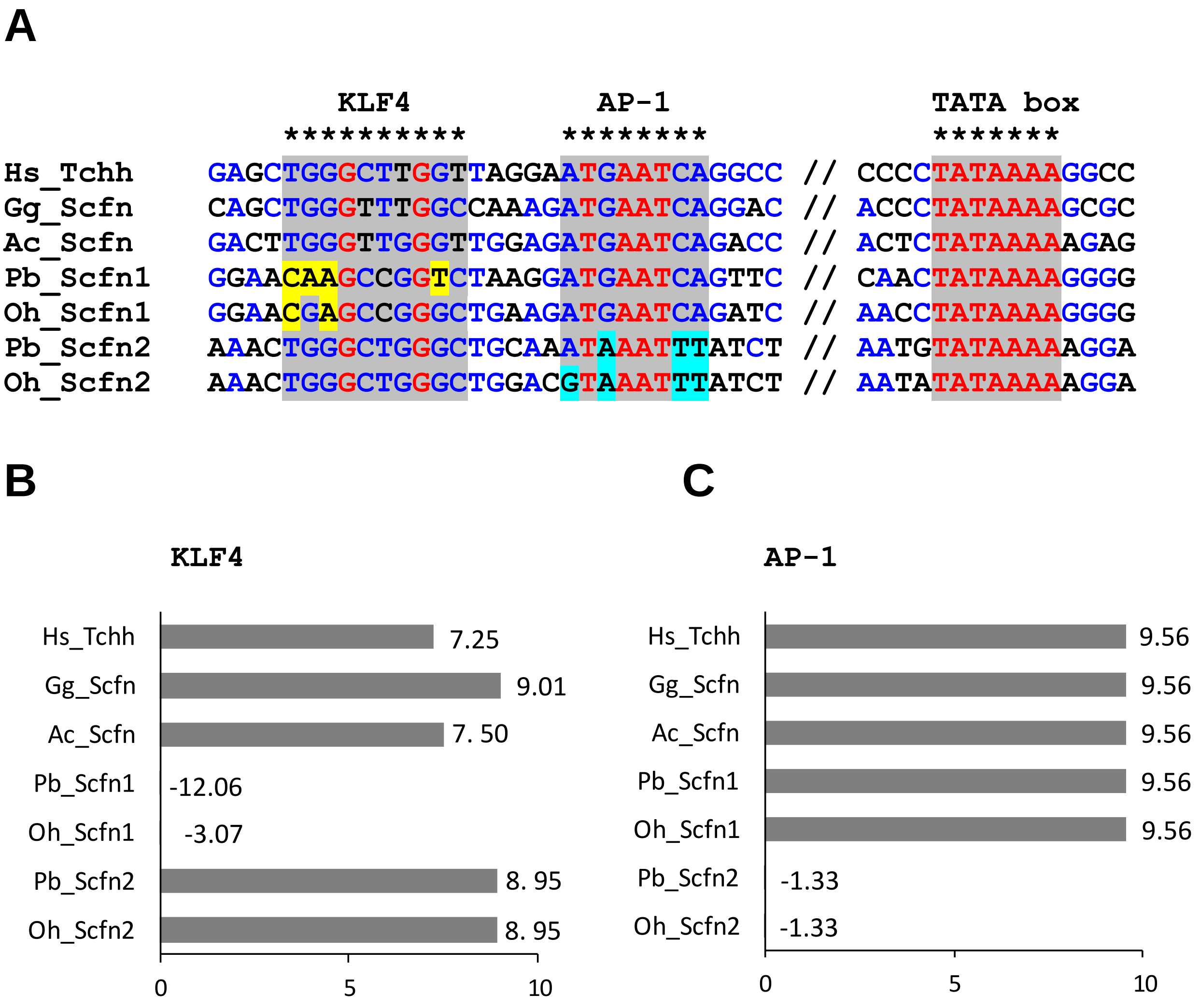

Suppl. Fig. S10. Transcription factor binding sites in the promoters of SFTP genes. (A) Nucleotide sequences of the proximal promoters of the genes encoding S100 fused-type proteins (SFTPs) were aligned. As a previous investigation, which did not include sequences of snakes, suggested the presence of binding sites for the transcription factors KLF4 and AP-1 in SFTP promoters (see Figure S4 in Mlitz et al. 2014), the transcription factor binding scores were calculated using the JASPAR 2016 server (http://jaspar.genereg.net). The positions of putative binding sites are indicated. Nucleotides compatible with the consensus binding motifs are shaded grey whereas nucleotides incompatible with the transcription factor binding are highlighted with yellow and blue background. **(B)** Binding scores for KLF4 in the promoters of SFTP genes at the site indicated in panel **A**. **(C)** Binding scores for AP-1 in the promoters of SFTP genes at the site indicated in panel **A**. Human (*Homo sapiens*, Hs), chicken (*Gallus gallus*, Gg), green anole lizard (*Anolis carolinensis*, Ac), python (*Python bivittatus*, Pb), and cobra (*Ophiophagus hannah*, Oh) sequences were analyzed. Scfn, scaffoldin; Tchh, trichohyalin.

Suppl. Table S1
Tentative abbreviations and full names of EDC genes identified in this study

Gene name abbreviation	Full gene name
Crnn	Cornulin
EDCATM	Epidermal Differentiation protein containing the CAT Motif
EDCG	Epidermal Differentiation protein rich in Cysteine and Glycine repeats
EDCM	Epidermal Differentiation protein containing a CCCC Motif
EDCP	Epidermal Differentiation protein rich in Cysteine and Proline
EDCRP	Epidermal Differentiation Cysteine-Rich Protein
EDCS1	Epidermal Differentiation protein, Cysteine-rich Short 1
EDCS2	Epidermal Differentiation protein, Cysteine-rich Short 2
EDCS3	Epidermal Differentiation protein, Cysteine-rich Short 3
EDEPK	Epidermal Differentiation protein rich in glutamic acid (E), Proline and lysine (K)
EDEPT	Epidermal Differentiation protein rich in glutamic acid (E), Proline and Threonine
EDETM	Epidermal Differentiation protein containing an ET Motif
EDETM2	Epidermal Differentiation protein containing an ET Motif 2
EDGPC	Epidermal Differentiation protein rich in Glycine, Proline and Cysteine
EDGPC2	Epidermal Differentiation protein rich in Glycine, Proline and Cysteine 2
EDGY1	Epidermal Differentiation protein rich in Glycine and tyrosine (Y) 1
EDGY2	Epidermal Differentiation protein rich in Glycine and tyrosine (Y) 2
EDHEM	Epidermal Differentiation protein containing a HEM Motif
EDKM	Epidermal Differentiation protein containing a KKLIQQ Motif
EDP3	Epidermal Differentiation protein rich in Proline 3
EDPAM	Epidermal Differentiation protein containing a PA Motif
EDPAML	Epidermal Differentiation protein containing a PA Motif Like
EDPCCC1	Epidermal Differentiation protein containing PCCC repeats 1
EDPCCC2	Epidermal Differentiation protein containing PCCC repeats 2
EDPCCC3	Epidermal Differentiation protein containing PCCC repeats 3
EDPCCC4	Epidermal Differentiation protein containing PCCC repeats 4
EDPCS	Epidermal Differentiation protein rich in Proline, Cysteine and Serine
EDPKC	Epidermal Differentiation protein rich in Proline, lysine (K) and Cysteine
EDPQ1	Epidermal Differentiation protein rich in Proline and glutamine (Q) 1
EDPQ2	Epidermal Differentiation protein rich in Proline and glutamine (Q) 2
EDPQ3	Epidermal Differentiation protein rich in Proline and glutamine (Q) 3
EDPS1	Epidermal Differentiation protein rich in Proline and Serine 1
EDPS2	Epidermal Differentiation protein rich in Proline and Serine 2
EDPS3	Epidermal Differentiation protein rich in Proline and Serine 3
EDPSQ	Epidermal Differentiation protein rich in Proline, Serine and glutamine (Q)
EDQK	Epidermal Differentiation protein containing glutamine (Q) and lysine (K) repeats
EDQL	Epidermal Differentiation protein rich in glutamine (Q), close to Loricrin
EDQL2	Epidermal Differentiation protein rich in glutamine (Q), close to Loricrin 2
EDQM	Epidermal Differentiation protein containing a glutamine (Q) Motif
EDQSG	Epidermal Differentiation protein rich in glutamine (Q), Serine and Glycine
EDSC1	Epidermal Differentiation protein rich in Serine and Cysteine 1
EDSC2	Epidermal Differentiation protein rich in Serine and Cysteine 2
EDSCP	Epidermal Differentiation protein rich in Serine, Cysteine and Proline
EDSPR1	Epidermal Differentiation protein Small Proline Rich 1
EDSPR2	Epidermal Differentiation protein Small Proline Rich 2
EDSQ	Epidermal Differentiation protein rich in Serine and glutamine (Q)
EDSRWM	Epidermal Differentiation protein containing a SRW Motif
EDWM	Epidermal Differentiation protein containing a WYDP Motif
EDY1	Epidermal Differentiation protein rich in tyrosine (Y)
EDYM1	Epidermal Differentiation protein containing Y Motif 1
EDYM2	Epidermal Differentiation protein containing Y Motif 2
Lor1	Loricrin 1
Lor2	Loricrin 2
Pglyrp3	Peptidoglycan recognition protein 3
Scfn1	Scaffoldin 1
Scfn2	Scaffoldin 2

Note - EDC genes encoding corneous beta-proteins (beta-keratins) and S100A proteins are not included here.

Suppl. Table S2
Burmese python *(Python bivittatus)* EDC genes (other than corneous beta protein genes)

Gene	Accession nr.	CDS start	CDS end	Sequence complete	Expression confirmed by *P. regius* RNA-seq data
S100A1	NW_006533184.1	1588	555	yes	yes
S100A13	NW_006533184.1	14911	15919	yes	yes
S100A14	NW_006533184.1	19278	23485	yes	yes
S100A16	NW_006533184.1	37282	38809	yes	yes
S100A2	NW_006533184.1	46616	47229	yes	yes
S100A3	NW_006533184.1	51063	52413	yes	yes
S100A4	NW_006533184.1	56042	57171	yes	yes
S100A5	NW_006533184.1	61202	61812	yes	yes
S100A6	NW_006533184.1	66291	66748	yes	yes
S100A12	NW_006539396.1	10278	9093	yes	yes
PGLYRP3	NW_006539396.1	4288	1166	yes	yes
EDKM	NW_006540970.1	21714	20641	yes	yes
EDPQ3	NW_006540970.1	15196	15360	yes	yes
EDPQ2	NW_006540970.1	9469	9227	yes	yes
EDSC1	NW_006543838.1	4076	3660	yes	no
EDSC2	NW_006543838.1	12950	13385	no	no
EDQM	NW_006533945.1	464	240	yes	yes
EDWM	NW_006533945.1	10373	9753	yes	yes
EDPQ1	NW_006533945.1	15513	14725	yes	yes
EDCS1	NW_006533945.1	21723	22019	yes	yes
EDHEM	NW_006533945.1	24848	24321	yes	yes
EDCS2	NW_006533945.1	31505	31266	yes	yes
EDCM	NW_006533945.1	35942	36157	yes	yes
EDCS3	NW_006533945.1	40476	39946	yes	yes
EDPCCC1	NW_006533945.1	46880	46335	yes	yes
EDPCCC2	NW_006533945.1	51062	50646	yes	yes
EDPCCC3	NW_006533945.1	63364	61049	no	yes
EDCG	NW_006533945.1	77683	77486	yes	yes
EDPCCC4	NW_006533945.1	81785	81345	yes	yes
EDGPC	NW_006533945.1	85309	85572	yes	yes
EDQL	NW_006533945.1	92571	<92239	no	yes
LOR2	NW_006533945.1	102481	101773	no	yes
LOR1	NW_006533945.1	109065	107293	no	no
EDY1	NW_006538280.1	8900	8205	yes	yes
EDSRWM	NW_006538280.1	15145	17079	yes	yes
EDGY1	NW_006538280.1	23044	22733	yes	yes
EDGY2	NW_006538280.1	34934	35329	yes	yes
EDETM	NW_006540169.1	33959	34210	yes	yes
EDPS1	NW_006541849.1	19165	20127	yes	yes
EDPS2	NW_006547155.1	<1262	867	no	no
EDPS3	NW_006533133.1	7040	7441	yes	yes
EDSCP	NW_006533133.1	11620	10994	yes	yes
EDYM2	NW_006533133.1	51320	50361	yes	yes
EDPSQ	NW_006533133.1	59918	58921	yes	yes
EDEPK	NW_006533133.1	66212	65754	yes	no
EDPKC	NW_006533133.1	77786	78652	yes	yes
EDP3	NW_006533133.1	83736	83521	yes	yes
EDQSG	NW_006533133.1	87350	87931	yes	yes
EDSPR1	NW_006533133.1	90689	90375	yes	yes
EDPCS	NW_006533133.1	97800	98342	yes	yes
EDCP	NW_006533133.1	107373	106795	yes	yes
EDCATM	NW_006533133.1	113018	113974	yes	yes
EDPAM	NW_006533133.1	121454	122536	yes	yes
EDSQ	NW_006533133.1	130252	131010	yes	yes
EDEPT	NW_006533133.1	135106	135657	yes	yes
EDSPR2	NW_006533133.1	139240	139043	yes	yes
CRNN	NW_006533133.1	146971	149243	yes	yes
SCFN2	NW_006533133.1	154919	152258	no	no
SCFN1	NW_006533133.1	160207	164241	yes	no
S100A11	NW_006533133.1	167015	170608	yes	yes
S100A10	NW_006533133.1	183743	184878	yes	yes

Notes - CDS, coding sequence.
The symbols < and > indicate that ends of the coding sequence were not present on the scaffold.

Suppl. Table S3

Burmese python *(Python bivittatus)* corneous beta protein (beta-keratin) genes

Gene	Accession nr.	CDS start	CDS end	Sequence complete	Expression confirmed by *P. regius* RNA-seq data
Beta1	NW_006533945.1	129573	128446	yes	yes
Beta2	NW_006533945.1	141471	141166	yes	yes
Beta3	NW_006533945.1	148257	149408	yes	yes
Beta4	NW_006533945.1	153207	152950	yes	yes
Beta5	NW_006533945.1	162249	161980	yes	yes
Beta6	NW_006533945.1	175889	175638	yes	no
Beta7	NW_006533945.1	178952	179227	yes	no
Beta8	NW_006533945.1	182958	182686	yes	yes
Beta9	NW_006533945.1	185558	185833	yes	no
Beta10	NW_006533945.1	187387	187124	yes	yes
Beta11	NW_006533945.1	193099	192722	yes	no
Beta12	NW_006533945.1	196728	197000	yes	no
Beta13	NW_006533945.1	204035	203727	yes	no
Beta14	NW_006533945.1	212727	213041	yes	yes
Beta15	NW_006538280.1	44064	44354	yes	no
Beta16	NW_006538280.1	49426	49821	yes	yes
Beta17	NW_006538280.1	52808	52377	yes	yes
Beta18	NW_006538280.1	57570	58020	yes	yes
Beta19	NW_006538280.1	62708	62235	yes	yes
Beta20	NW_006542926.1	15275	>15577	no	yes
Beta21	NW_006542926.1	2255	2746	yes	yes
Beta22	NW_006551345.1	>212	15	no	no
Beta23	NW_006555159.1	>522	334	no	no
Beta24	NW_006552039.1	197	556	yes	no
Beta25	NW_006540169.1	583	1002	yes	yes
Beta26	NW_006540169.1	5880	<5596	no	yes
Beta27	NW_006540169.1	<11505	11825	no	yes
Beta28	NW_006540169.1	17257	16829	yes	yes
Beta29	NW_006541849.1	>5559	5290	no	yes
Beta30	NW_006541849.1	13806	13432	yes	yes
Beta31	NW_006533133.1	21000	20548	yes	yes
Beta32	NW_006533133.1	27649	28053	yes	yes
Beta33	NW_006533133.1	31407	30952	yes	yes
Beta34	NW_006533133.1	39313	39747	yes	no
Beta35	NW_006533133.1	43812	43522	yes	no

Notes - CDS, coding sequence.

The symbols < and > indicate that ends of the coding sequence were not present on the scaffold.

Suppl. Table S4
King cobra (*Ophiophagus hannah*) EDC genes (other than corneous beta protein genes)

Gene	Accession nr.	CDS start	CDS end	Sequence complete
S100A9	AZIM01042421.1	45	>188	no
PGLYRP3	AZIM01003248.1	1855	5074	yes
EDKM	AZIM01003248.1	12034	12171	yes
EDPQ3	AZIM01003248.1	19465	<19349	no
EDPQ2	AZIM01003248.1	33024	33395	yes
EDSC1	AZIM01003248.1	41385	41068	yes
EDSC2	AZIM01003248.1	52467	52712	yes
EDWM	AZIM01003248.1	68925	68212	yes
EDPQ1	AZIM01003248.1	73668	72802	yes
EDCS1	AZIM01003248.1	78687	78968	yes
EDHEM	AZIM01003248.1	82623	82117	yes
EDCS2	AZIM01003248.1	87881	87576	yes
EDCM	AZIM01003248.1	92670	92894	yes
EDCS3	AZIM01003248.1	97472	96797	yes
EDPCCC1	AZIM01003248.1	104405	104049	yes
EDPCCC2	AZIM01003248.1	109843	109472	yes
EDPCCC3	AZIM01003248.1	122470	118393	no
EDCG	AZIM01003248.1	133443	133243	yes
EDPCCC4	AZIM01003248.1	137976	137235	no
EDGPC	AZIM01003248.1	141722	142009	yes
EDQL	AZIM01003248.1	145280	144999	yes
LOR2	AZIM01003248.1	151769	150621	yes
LOR1	AZIM01003248.1	157066	155969	yes
EDY1	AZIM01004187.1	73541	74254	yes
EDSRWM	AZIM01004187.1	64099	62798	yes
EDGY1	AZIM01004187.1	58166	58483	yes
EDGY2	AZIM01004187.1	47891	47478	yes
EDETM	AZIM01000954.1	23704	23952	yes
EDPS1	AZIM01000954.1	41059	41844	yes
EDPS2	AZIM01000954.1	58107	58886	yes
EDPS3	AZIM01000954.1	<63568	63978	no
EDSCP	AZIM01000954.1	69647	69066	yes
EDYM2	AZIM01000954.1	107053	106094	yes
EDEPK	AZIM01000954.1	119831	119364	yes
EDPKC	AZIM01000954.1	131139	131972	yes
EDP3	AZIM01000954.1	135690	135409	yes
EDQSG	AZIM01000954.1	140286	140879	yes
EDSPR1	AZIM01000954.1	146227	145952	yes
EDPCS	AZIM01000954.1	153869	154345	yes
EDQK	AZIM01000954.1	156359	156141	yes
EDCP	AZIM01000954.1	162072	161572	yes
EDCATM	AZIM01000954.1	167242	168537	yes
EDPAM	AZIM01000954.1	174551	175153	yes
EDSQ	AZIM01000954.1	183997	184629	yes
EDEPT	AZIM01004605.1	3309	3890	yes
EDSPR2	AZIM01004605.1	8254	8066	yes
CRNN	AZIM01004605.1	14916	17816	yes
SCFN2	AZIM01004605.1	23685	21601	yes
SCFN1	AZIM01004605.1	28678	32401	yes
S100A11	AZIM01004605.1	<38940	40166	no

Notes - CDS, coding sequence. Further S100A genes are present close to border of the EDC.
The symbols < and > indicate that ends of the coding sequence were not present on the scaffold.

Suppl. Table S5
King cobra (*Ophiophagus hannah*) corneous beta protein (beta-keratin) genes

Gene	Accession nr.	CDS start	CDS end	Sequence complete
Beta1	AZIM01003248.1	174867	173737	yes
Beta2	AZIM01003044.1	87502	87801	yes
Beta3	AZIM01003044.1	81345	79850	yes
Beta4	AZIM01003044.1	76531	76767	yes
Beta5	AZIM01003044.1	68820	69074	yes
Beta6	AZIM01003044.1	55588	55848	yes
Beta7	AZIM01003044.1	52619	52344	yes
Beta8	AZIM01003044.1	49125	49403	yes
Beta9	AZIM01003044.1	43757	43984	yes
Beta10	AZIM01007131.1	26847	27329	yes
Beta11	AZIM01007131.1	17992	17738	yes
Beta12	AZIM01007131.1	13225	13509	yes
Beta13	AZIM01007131.1	3381	3070	yes
Beta14	AZIM01004187.1	42024	41680	yes
Beta15	AZIM01004187.1	36898	36512	yes
Beta16	AZIM01004187.1	34624	35034	yes
Beta17	AZIM01004187.1	30955	30533	yes
Beta18	AZIM01004187.1	27140	27531	no
Beta19	AZIM01004187.1	23240	22722	yes
Beta20	AZIM01004187.1	18585	18992	yes
Beta21	AZIM01004187.1	7539	7093	yes
Beta22	AZIM01004187.1	2467	2882	no
Beta23	AZIM01041946.1	772	344	yes
Beta24	AZIM01008786.1	16443	16057	yes
Beta25	AZIM01008286.1	17037	16576	yes
Beta26	AZIM01008286.1	11937	12416	yes
Beta27	AZIM01008286.1	8808	8353	yes
Beta28	AZIM01008286.1	1958	>2062	no
Beta29	AZIM01000954.1	3170	2733	yes
Beta30	AZIM01000954.1	29658	29272	yes
Beta31	AZIM01000954.1	38016	37636	yes
Beta32	AZIM01000954.1	47356	46940	yes
Beta33	AZIM01000954.1	76707	76306	yes
Beta34	AZIM01000954.1	85348	85815	yes
Beta35	AZIM01000954.1	89721	89242	yes
Beta36	AZIM01000954.1	100589	101041	yes

Notes - CDS, coding sequence.

The symbols < and > indicate that ends of the coding sequence were not present on the scaffold.

Suppl. Table S6
Green anole lizard *(Anolis carolinensis)* EDC genes newly (*) identified in the present study.

Gene	Accession nr.	CDS start	CDS end	Sequence complete	Expression confirmed by RNA-seq data (*A. carolinensis*)
EDSC2	NW_003338916.1	1040943	1040629	yes	yes
EDQM	NW_003338916.1	1036851	1037078	yes	yes
EDHEM	NW_003338916.1	995360	996070	yes	yes
EDCS1	NW_003338916.1	990209	990406	yes	yes
EDCS2	NW_003338916.1	982933	983241	yes	yes
EDCS3	NW_003338916.1	961905	962762	yes	yes
EDPCCC1	NW_003338916.1	951749	951994	yes	yes
EDPCCC2	NW_003338916.1	936661	936473	yes	yes
EDPCCC3	NW_003338916.1	<919157	919798	no	yes
EDGPC1	NW_003338916.1	893728	893540	yes	yes
EDGPC2	NW_003338916.1	876803	876600	yes	yes
EDYM1	NW_003338916.1	780003	780617	yes	yes
EDY1	NW_003338916.1	571522	572292	yes	yes
EDSRWM	NW_003338916.1	561996	560887	yes	yes
EDGY1	NW_003338916.1	556637	557008	yes	yes
EDGY2	NW_003338916.1	540103	539129	yes	yes
EDETM1	NW_003338916.1	305599	305339	yes	yes
EDETM2	NW_003338916.1	298118	297858	yes	yes
EDPCCC4	NW_003338916.1	238742	239044	yes	no
EDYM2	NW_003338916.1	184370	185365	yes	yes
EDP3	NW_003338916.1	129131	129376	yes	yes
EDQSG	NW_003338916.1	120578	120174	yes	yes
EDQK	NW_003338916.1	87721	87867	yes	yes
EDCATM	NW_003338916.1	57594	57070	yes	yes
EDPAML	NW_003338916.1	47259	46576	yes	yes

Notes - * other EDC genes have been reported in a previous paper of our laboratories (30).

CDS, coding sequence; the symbols < and > indicate that ends of the coding sequence were not present on the scaffold.
